# Initial biomarker testing strategies and clinical outcomes in advanced non-small cell lung cancer

**DOI:** 10.1016/j.isci.2026.115966

**Published:** 2026-05-15

**Authors:** Masaki Ishida, Tadaaki Yamada, Yasuhiro Goto, Taiichiro Otsuki, Hirokazu Taniguchi, Taishi Harada, Akihiro Yoshimura, Shinsuke Shiotsu, Asuka Okada, Kazuki Jinno, Hibiki Kanda, Noeru Inoguchi, Mototaka Fukui, Takahiro Yamada, Tae Hata, Hayato Kawachi, Yuki Katayama, Kenji Morimoto, Masahiro Iwasaku, Takashi Kijima, Koichi Takayama

**Affiliations:** 1 Department of Pulmonary Medicine, Graduate School of Medical Science, Kyoto Prefectural University of Medicine, Kajii-cho, Kamigyo-ku, Kyoto, Japan; 2 Department of Respiratory Medicine, Fujita Health University School of Medicine, Toyoake, Aichi, Japan; 3 Department of Respiratory Medicine and Hematology, School of Medicine, Hyogo Medical University, Nishinomiya, Hyogo, Japan; 4 Department of Respiratory Medicine, Nagasaki University Graduate School of Biomedical Sciences, Nagasaki, Japan; 5 Department of Medical Oncology, Fukuchiyama City Hospital, Fukuchiyama, Kyoto, Japan; 6 Department of Respiratory Medicine, Japanese Red Cross Kyoto Daini Hospital, Kyoto, Japan; 7 Department of Respiratory Medicine, Saiseikai Suita Hospital, Suita, Osaka, Japan; 8 Department of Respiratory Medicine, Japanese Red Cross Kyoto Daiichi Hospital, Kyoto, Japan; 9 Department of Respiratory Medicine, Omi Medical Center, Kusatsu, Shiga, Japan; 10 Department of Pulmonary Medicine, Otsu City Hospital, Otsu, Shiga, Japan; 11 Department of Respiratory Medicine, Uji-Tokushukai Medical Center, Kyoto, Japan; 12 Department of Pulmonary Medicine, Matsushita Memorial Hospital, Osaka, Japan

**Keywords:** Health sciences

## Abstract

Appropriate initial biomarker testing is essential for treatment selection in patients with advanced non-small cell lung cancer (NSCLC). We conducted a multicenter retrospective study comparing single-plex and multiplex testing strategies for driver oncogene detection and clinical outcomes. Single-plex testing was associated with a higher detection rate of epidermal growth factor receptor (*EGFR*) mutations, greater use of *EGFR* tyrosine kinase inhibitors, and longer overall survival than multiplex testing. These findings suggest that limitations in the sensitivity of currently available multiplex platforms may influence downstream treatment opportunities, particularly in populations with a high prevalence of *EGFR* mutations. Our results support further optimization of multiplex assays and evaluation of sequential testing strategies for patients without detected driver oncogenes on initial testing.

## Highlights

Single-plex testing detected more *EGFR* mutations than multiplex testing
• Single-plex testing was linked to longer overall survival
• Overall driver detection was similar between testing strategies
• Results support optimization of multiplex assays and sequential testing

## Graphical abstract

**Figure:**
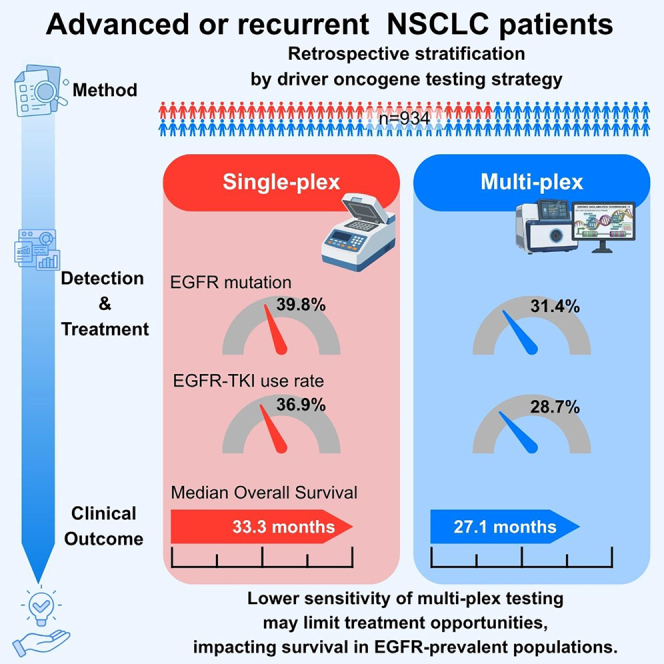

## Introduction

Lung cancer is the primary cause of cancer-related death worldwide.^1^ Non-small cell lung cancer (NSCLC) is the most common lung cancer subtype, accounting for approximately 85% of all reported lung cancer cases.^2^ The identification of driver oncogenes is among the most significant advancements in the field of lung cancer over the past two decades. The subsequent development of targeted therapies has markedly improved the prognosis for patients with advanced NSCLC.^3^^,^^4^^,^^5^^,^^6^^,^^7^ In addition, numerous studies have demonstrated that patients with detected driver oncogenes who were treated with molecularly targeted agents against these oncogenes achieved prolonged survival and improved prognosis.^8^^,^^9^ Multiple biomarker testing for patients with advanced NSCLC, including epidermal growth factor receptor (*EGFR* [OMIM 131550]) mutations, anaplastic lymphoma kinase (*ALK* [OMIM 105590]) fusion, *ROS1* (ROS1 [OMIM 165020]) fusion, *BRAF* V600E (*BRAF* [OMIM 164757]) mutations, *MET* (*MET* [OMIM 164860]) exon 14 skipping mutations, rearranged during transfection (*RET* [OMIM 164761]) fusion, kirsten rat sarcoma virus (*KRAS* [OMIM 190070]) G12C mutations, human epidermal growth factor receptor type 2 (HER2 [OMIM 164870]) mutations, and neurotrophic tropomyosin receptor kinase (*NTRK* [OMIM 191315, 600456, and 191316]) fusion, is currently strongly recommended to detect these driver oncogenes.^10^^,^^11^^,^^12^^,^^13^ The diagnostic methods for driver oncogenes in patients with advanced NSCLC can be evaluated using either single-plex or multiplex testing. Single-plex testing evaluates only a single driver oncogene, whereas multiplex testing simultaneously evaluates multiple driver oncogenes in a single assay. The number of driver oncogenes that need to be evaluated in patients with advanced NSCLC is increasing. Consequently, routine clinical practice has seen a rapid shift from conventional single-plex to multiplex testing, which can shorten the turnaround time (TAT) to diagnosis and cover a wide range of driver oncogenes.^12^^,^^14^^,^^15^^,^^16^

The proportion of *EGFR* mutations in lung cancer is considerably higher in Asian than that in Western populations. In Japan, the prevalence of *EGFR* mutations in NSCLC is estimated to be 45% (range, 21%–68%).^17^ However, *EGFR* mutations have been detected in less than 30% of the patients using multiplex testing.^18^^,^^19^ Therefore, the observed proportion of *EGFR* mutations may vary depending on the type of biomarker testing employed. Additionally, a recent retrospective data collection study reported that the implementation rate of multiplex testing in patients with advanced NSCLC is 47.7% in clinical practice.^8^ The existence of such differences in biomarker testing could potentially influence several variables, including the proportion of detected driver oncogenes, the number of patients receiving appropriate molecular targeted therapies, and ultimately the clinical outcomes of patients with advanced non-squamous NSCLC. However, no large-scale cohort study has yet evaluated the impact of differences in biomarker testing strategies performed at the time of initial treatment on clinical outcomes.^20^^,^^21^ Therefore, we conducted this multicenter retrospective study to evaluate the impact that biomarker testing strategies have on clinical outcomes in patients with advanced non-squamous NSCLC.

## Results

### Patient characteristics and sample condition

**Figure 1:**
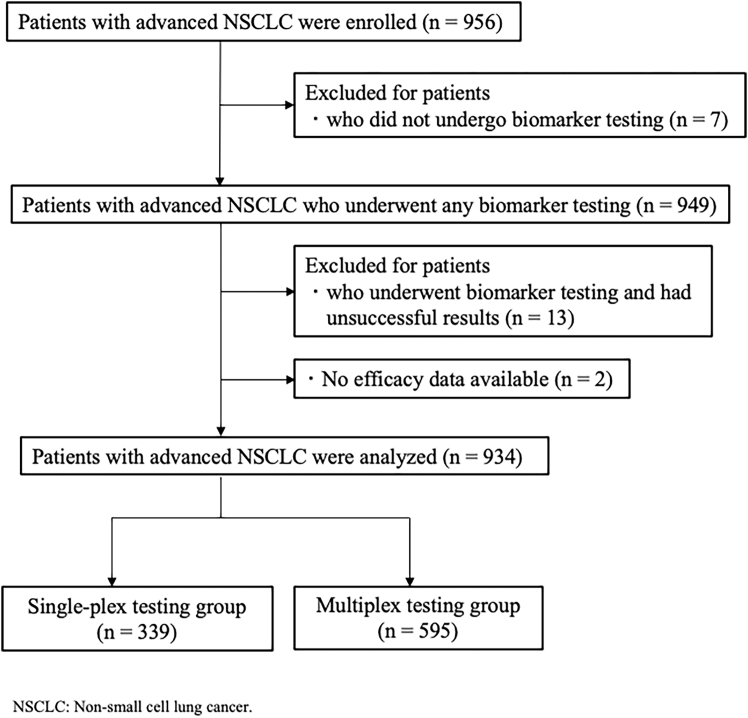
Consort diagram of this study

Our study included 956 patients with advanced non-squamous NSCLC, among which seven did not undergo biomarker testing, leaving 949 patients eligible for molecular evaluation. Of these patients, 13 (1.4%) experienced assay failure and were excluded. Two patients were excluded because efficacy data were unavailable. Finally, 934 patients were included in the analysis (Figure 1). Single-plex and multiplex testing were performed in 339 (36.3%) and 595 (63.7%) patients, respectively. Among the 12 participating institutions, 11 had experience in performing both testing methods. Multiplex testing was the predominant approach used in eight of these institutions, accounting for more than half of all biomarker testing performed (Table S1A). The distribution of assay types used in each test is shown in Tables S1B and S1C, demonstrating that cobas *EGFR* mutation test v2 was predominant in the single-plex group (*n* = 323/339, 95.3%), whereas the Oncomine Dx target test was mainly used in the multiplex group (*n* = 434/595, 72.9%).

Baseline patient and sample characteristics are summarized in Table 1. In the single-plex group, the median age was 72 years (range, 32–93), 38.6% of patients were female, and 94.1% had an eastern cooperative oncology group performance status (ECOG-PS) of 0–1. Driver oncogenes were detected in 45.4% of patients, and 42.5% received molecularly targeted therapy at any line. In the multiplex group, the median age was 72 years (range, 30–93 years), 34.1% were female, and 90.1% had an ECOG-PS of 0–1. Driver oncogenes were detected in 51.1% of patients, and 38.8% received molecularly targeted therapy at any line. Patients who underwent single-plex testing had a better PS than those who underwent multiplex testing did (*p* = 0.04). No other significant differences in baseline patient characteristics were observed between the two groups. Similarly, no significant differences were observed between the single-plex and multiplex testing groups in the proportions of tissue and cytology specimens (tissue: 93.8% vs. 95.6%; cytology: 6.2% vs. 4.4%, *p* = 0.28) or in the distribution of samples obtained from primary versus metastatic lesions (primary: 68.7% vs. 71.8%; metastatic: 31.3% vs. 28.2%, *p* = 0.36). The median TAT was shorter in the single-plex group than in the multiplex group (9 vs. 11 days, *p* < 0.001). To evaluate whether TAT influenced the timing of treatment initiation, we assessed the correlation between TAT and the interval from diagnosis to initiation of first-line therapy. A weak but statistically positive correlation was observed (Spearman *r* = 0.135, 95% confidence intervals [CIs]: 0.062–0.208, *p* < 0.001) (Figure S1). Empirical systemic therapy initiated before receipt of biomarker test results was less frequent in the single-plex group than in the multiplex group (4.7% vs. 8.6%, *p* = 0.03).

**Table 1:** Patient and sample characteristics (*n* = 934)

Characteristics	All patients (*n* = 934)	Single-plex testing group (*n* = 339)	Multiplex testing group (*n* = 595)	*p* value
Age				
Median (range)	72 (30–93)	72 (32–93)	72 (30–93)	0.53
≥75	336 (36.0%)	121 (35.7%)	215 (36.1%)	0.94
Sex				
Female	334 (35.8%)	131 (38.6%)	203 (34.1%)	0.18
ECOG-PS				
0–1	855 (91.5%)	319 (94.1%)	536 (90.1%)	**0.04**
2–4	70 (7.5%)	19 (5.6%)	51 (8.6%)	
Missing data	9 (1.0%)	1 (0.3%)	8 (1.3%)	
Tumor histology				
Adenocarcinoma	800 (85.7%)	289 (85.3%)	511 (85.9%)	0.85
Other	134 (14.3%)	50 (14.7%)	84 (14.1%)	
Stage				
Ⅲ–IV	776 (83.1%)	289 (85.3%)	487 (81.8%)	0.20
Postoperative Recurrence	158 (16.9%)	50 (14.7%)	108 (18.2%)	
Metastatic site				
Extrathoracic	576 (61.7%)	201 (59.3%)	375 (63.0%)	0.29
Bone	314 (33.6%)	109 (32.3%)	205 (34.5%)	0.50
Brain	228 (24.4%)	75 (22.1%)	153 (25.8%)	0.24
Liver	113 (12.1%)	34 (10.0%)	79 (13.3%)	0.13
PD-L1 TPS				
≥50%	279 (29.9%)	103 (30.4%)	176 (29.6%)	0.82
1–49%	304 (32.5%)	91 (26.8%)	213 (35.8%)	
<1%	290 (31.0%)	106 (31.3%)	184 (30.9%)	
Unknown	61 (6.5%)	39 (11.5%)	22 (3.7%)	
Smoking status				
Current/Former	596 (63.8%)	208 (61.4%)	388 (65.2%)	0.26
Never	325 (34.8%)	124 (36.6%)	201 (33.8%)	
Missing data	13 (1.4%)	7 (2.1%)	6 (1.0%)	
Driver oncogenes detected				
Yes	458 (49.0%)	154 (45.4%)	304 (51.1%)	0.10
Molecularly targeted therapy at any line	375 (40.1%)	144 (42.5%)	231 (38.8%)	0.27
Type of testing				
Tissue	887 (95.0%)	318 (93.8%)	569 (95.6%)	0.28
Cytology	47 (5.0%)	31 (6.2%)	26 (4.4%)	
Site of sample				
Primary lesion	660 (70.7%)	233 (68.7%)	427 (71.8%)	0.36
Metastatic lesion	274 (29.3%)	106 (31.3%)	168 (28.2%)	
Turnaround time (day)				
Median (IQR)	11 (6–15)	9 (6–14)	11 (8–15)	**<0.001**
Empirical systemic therapy before receipt of biomarker status				
Yes	67 (7.2%)	16 (4.7%)	51 (8.6%)	**0.03**

ECOG-PS: eastern cooperative oncology group performance status, PD-L1 TPS: programmed death ligand 1 tumor proportion score, ICI: immune checkpoint inhibitor. Bold values indicate statistically significant differences (*p* < 0.05).

### Prevalence of driver oncogenes and use of targeted and immune-based therapies

**Figure 2:**
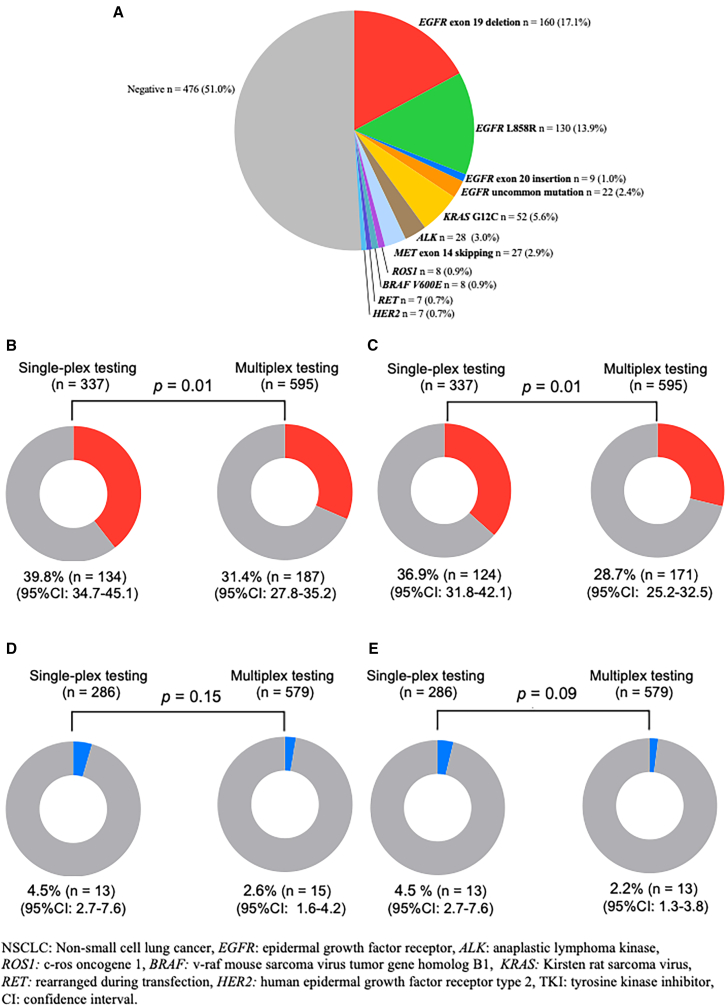
Distribution of driver oncogenes and administration of targeted therapies according to biomarker testing strategy (A–E) Proportion of (A) driver oncogenes in all patients with non-squamous NSCLC (*n* = 934), (B) EGFR mutations, (C) patients who received EGFR-TKIs, (D) ALK fusions, and (E) patients who received ALK-TKIs, according to the type of biomarker testing.

Among the detected driver oncogenes, *EGFR* mutations were the most prevalent (34.4%, *n* = 321), including exon 19 deletions (17.1%, *n* = 160), L858R mutations (13.9%, *n* = 130), exon 20 insertions (1.0%, *n* = 9), and uncommon mutations (2.4%, *n* = 22). *KRAS* G12C mutation was the second most prevalent driver oncogene (5.6%, *n* = 52). The other identified driver oncogenes were *ALK* fusions (3.0%, *n* = 28), *MET* exon 14 skipping mutations (2.9%, *n* = 27), *ROS1* fusions (0.9%, *n* = 8), *BRAF* V600E mutations (0.9%, *n* = 8), *RET* fusions (0.7%, *n* = 7), and *HER2* mutations (0.7%, *n* = 7) (Figure 2A). Subsequently, we compared driver oncogene distributions according to the type of testing. In the single-plex group, *EGFR* mutations were detected in 39.3% of patients, including exon 19 deletions (20.1%, *n* = 68), L858R mutations (15.3%, *n* = 52), exon 20 insertions (1.5%, *n* = 5), and uncommon mutations (2.7%, *n* = 9) (Figure S2A). In the multiplex group, *EGFR* mutations were detected in 31.5% of patients, including exon 19 deletions (15.5%, *n* = 92), L858R mutations (13.1%, *n* = 78), exon 20 insertions (0.7%, *n* = 4), and uncommon mutations (2.2%, *n* = 13) (Figure S2B).

Among the patients who underwent *EGFR* mutation testing in each group, single-plex testing showed a significantly higher *EGFR* mutations detection rate than that of multiplex testing (39.8% vs. 31.4%, *p* = 0.01) (Figure 2B). In addition, the proportion of patients who received EGFR-TKIs was significantly higher in the single-plex testing group than in the multiplex testing group (36.9% vs. 28.7%, *p* = 0.01) (Figure 2C). As shown in Table S2, the use of osimertinib was higher in the single-plex group (31.3% vs. 24.9%, *p* = 0.04), whereas the use of EGFR-TKIs, excluding osimertinib, did not differ significantly between the two groups (14.5% vs. 13.5%, *p* = 0.38). The proportion of patients who received immune checkpoint inhibitor (ICI)-containing regimens as first-line therapy was significantly lower in the single-plex group than in the multiplex group (42.8% vs. 53.4%, *p* = 0.002). Among the ICI-based regimens, programmed cell death protein 1/programmed death-ligand 1 (PD-1/PD-L1) inhibitor plus chemotherapy was the most frequently used approach (31.3% vs. 29.7%), followed by PD-1/CTLA-4 inhibitor plus chemotherapy (0.6% vs. 6.4%) and PD-1/PD-L1 plus anti-VEGF antibody regimens (1.2% vs. 5.4%). To confirm that these differences were not attributable to institutional heterogeneity, we compared *EGFR* mutation positivity rates across the 12 participating institutions. As shown in Figure S3A, *EGFR* positivity rates were consistent across institutions, ranging from 30.1% to 37.0%, indicating no major institutional bias in *EGFR* detection. The distribution of *EGFR* mutation subtypes according to testing platforms is summarized in Figure S3B. Across the testing platforms, *EGFR* exon 19 deletions and L858R mutations consistently represented the predominant *EGFR* mutation subtypes. Although overall *EGFR* detection rates varied across platforms (range, 19.0%–50.0%), no marked platform-specific bias was observed in the relative distribution of *EGFR* mutation subtypes. An analysis of annual trends from 2019 to 2022 demonstrated that the higher *EGFR* mutation detection rate in the single-plex testing group was consistently observed across all study years (Figure S4A). Similarly, the annual proportion of patients who received osimertinib remained higher in the single-plex group throughout the study period (Figure S4B). In contrast, no significant difference was observed between the detection rates of *ALK* fusions in the single-plex and multiplex groups (4.5% vs. 2.6%, *p* = 0.15) among patients who underwent *ALK* fusion in each testing (Figure 2D). The proportion of patients who received ALK-TKIs tended to be higher in those who underwent single-plex testing than that in those who underwent multiplex testing (4.5% vs. 2.2%, *p* = 0.09) (Figure 2E). The proportions of detected driver oncogenes other than *EGFR* and *ALK*, and the proportions of patients who received molecularly targeted therapy at any line, excluding EGFR- and ALK-targeted therapies, were significantly lower in the single-plex testing group than in the multiplex testing group (2.1% vs. 17.1%, *p* < 0.001 and 1.5% vs. 6.6%, *p* < 0.001, respectively) (Figures S5A and S5B). The proportions of patients who received matched targeted therapy for each driver alteration are shown in Figure S5C, excluding *EGFR* and *ALK* alterations. Matched targeted therapy was administered in 100% of patients with *ROS1* fusions (*n* = 8/8), followed by 87.5% with *BRAF* V600E mutation (*n* = 7/8), 71.4% with *RET* fusion (*n* = 5/7), 59.3% with *MET* exon 14 skipping (*n* = 16/27), 14.3% with *HER2* mutation (*n* = 1/7), and 13.5% with *KRAS* G12C mutation (*n* = 7/52). The number of patients with driver oncogenes excluding *EGFR* and *ALK* who were diagnosed after the approval of the corresponding targeted therapies and the proportion of patients who received matched treatments are summarized in Table S3. For example, among 23 patients with *KRAS* G12C mutations in the multiplex group diagnosed after January 2022, seven patients (30.4%) received sotorasib. To further minimize potential selection bias between testing groups, propensity score matching (PSM) analysis was performed, yielding 339 matched patients in each group with no significant differences in baseline characteristics (Table S4). After PSM adjustment, the proportion of patients harboring *EGFR* mutations and receiving EGFR-TKIs tended to be higher in the single-plex testing group than in the multiplex testing group (39.5% vs. 32.4%, *p* = 0.06, and 36.6% vs. 29.2%, *p* = 0.05, respectively) (Figures S6A and S6B).

### Impact of testing methods on treatment efficacy

**Figure 3:**
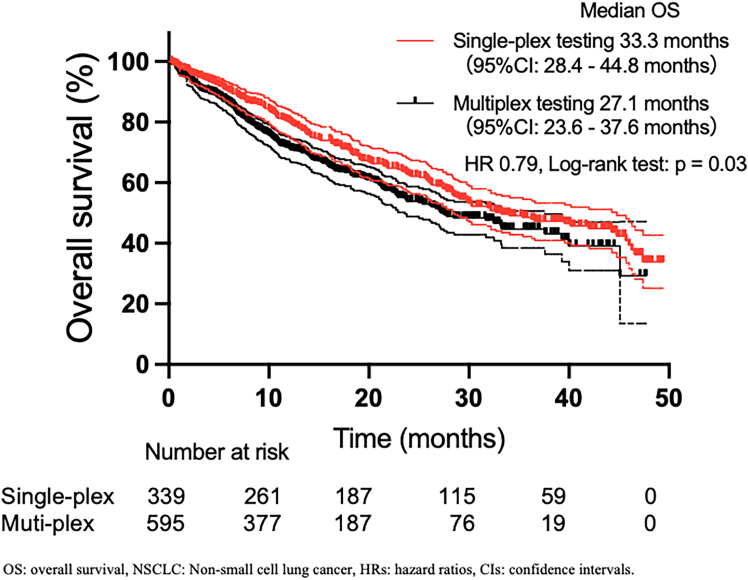
Kaplan-Meier curves for the OS of patients with advanced non-squamous NSCLC, according to the type of biomarker testing OS was estimated using the Kaplan–Meier method and compared between the single-plex and multiplex testing groups using the log rank test. Median OS values with 95% CIs are shown in the plot. HRs were estimated using a univariate Cox proportional hazards model.

The median overall survival (OS) of all patients with non-squamous NSCLC who underwent biomarker testing was 30.0 months (95% CIs, 27.2–35.4) (Figure S7). When stratified by testing modality, the patients who underwent single-plex testing exhibited a significantly longer OS than those who underwent multiplex testing did (33.3 vs. 27.1 months, log rank *p* = 0.03) (Figure 3). We subsequently performed exploratory subgroup analyses stratified by ECOG-PS, disease stage, and PD-L1 expression to assess whether the association between biomarker testing modality and OS was consistent across clinical subgroups. Hazard ratios (HRs) and 95% CIs derived from univariate Cox proportional hazards models are summarized as a forest plot in Figure S8. In patients with ECOG-PS 0–1, single-plex testing was associated with a longer OS, whereas no significant association was observed in patients with ECOG-PS 2–4. Similarly, in patients with stage III or IV disease, single-plex testing was associated with a longer OS, whereas no significant association was observed in patients with recurrent disease. When stratified by PD-L1 expression, a significant association favoring single-plex testing was observed in patients with PD-L1 1%–49%, whereas no significant association was observed in patients with PD-L1 ≥ 50% or <1%.

We then evaluated OS according to *EGFR* mutation status because differences in *EGFR* mutation detection may underlie the observed OS difference. Among patients evaluated for *EGFR* mutations, those who underwent single-plex testing had a longer OS than those who underwent multiplex testing did (33.2 vs. 27.2 months, log rank *p* = 0.03) (Figure S9A). Even after excluding patients harboring *EGFR* mutations, OS remained significantly longer in the single-plex testing group than in the multiplex testing group (28.6 vs. 21.3 months, log rank *p* = 0.02) (Figure S9B). We subsequently examined whether differences in treatment selection contributed to the OS differences observed between the two testing strategies. Among patients who received ICI-containing regimens as first-line therapy, those who underwent single-plex testing demonstrated longer OS than those who underwent multiplex testing did (29.8 vs. 21.2 months, log rank *p* = 0.04) (Figure S9C). Baseline characteristics of patients who received ICI-containing regimens were comparable between testing groups, except for the proportion of detected driver oncogenes (Table S5). In contrast, no significant OS difference was observed between testing modalities among patients who received molecular targeted therapy at any line (45.9 vs. 40.0 months, log rank *p* = 0.66) (Figure S9D). Similarly, testing modality did not influence OS among patients who received EGFR-TKIs as first-line therapy (44.8 vs. 40.0 months, log rank *p* = 0.84) (Figure S10A). When stratified by EGFR-TKI type, no significant OS differences were observed between single-plex and multiplex testing among patients treated with osimertinib (46.0 vs. 39.3 months, log rank *p* = 0.70) or among those who received EGFR-TKIs excluding osimertinib (27.7 months vs. not reached, log rank *p* = 0.26) (Figures S10B and S10C). Among the patients who received first- or third-generation EGFR-TKIs, the OS was longer in those treated with third-generation EGFR-TKIs (median 44.8 vs. 26.8 months; HR, 0.44; log rank *p* = 0.01) (Figure S10D). In the single-plex group, the OS was longer with third-generation EGFR-TKIs than with first-generation EGFR-TKIs (median 46.0 vs. 13.8 months; HR, 0.38; log rank *p* = 0.0002) (Figure S10E). In the multiplex group, the OS did not differ between first- and third-generation EGFR-TKIs (median 39.3 vs. 27.2 months; HR, 0.70; log rank *p* = 0.50) (Figure S10F). Taken together, these findings suggest that the observed OS difference between testing modalities was not observed among patients who received EGFR-TKIs and was primarily observed among patients who did not receive EGFR-TKIs.

Next, we evaluated outcomes in patients harboring actionable driver alterations, excluding *EGFR* or *ALK*. In this subgroup, the median OS was 20.3 months (95% CIs, 14.3-not reached) (Figure S11A). Patients who received matched targeted therapy demonstrated a markedly longer OS than those who did not (not reached vs. 15.3 months; HR, 0.39; log rank *p* = 0.004) (Figure S11B). To minimize potential confounding, PSM analysis was additionally performed. After PSM, patients who underwent single-plex testing continued to show significantly longer OS than those who underwent multiplex testing did (33.2 vs. 27.1 months; log rank *p* = 0.03) (Figure S6C). Finally, we conducted multivariable Cox regression analyses to confirm whether testing type independently predicted the OS. The Cox proportional hazards model for OS in patients with non-squamous NSCLC who underwent biomarker testing is presented in Table 2A. The proportional hazards assumption was assessed using Schoenfeld residuals and was not violated at the global level (*p* = 0.11). In the multivariable Cox model, evaluation of driver oncogenes using single-plex testing was independently associated with an improved OS (HR, 0.74; 95% CI, 0.59–0.93; *p* = 0.04). To assess the robustness of this finding, a sensitivity analysis was performed using a multivariable Cox proportional hazards model adjusting for between-institution differences. In this sensitivity analysis, the evaluation of driver oncogenes using single-plex testing remained independently and significantly associated with an improved OS (HR, 0.79; 95% CIs, 0.63–0.99; *p* = 0.04) (Table S6). To assess whether the prognostic impact of testing modality differed according to *EGFR* mutation status, we fitted an additional Cox model that included an interaction term between testing modality and *EGFR* mutation status. The interaction was not statistically significant (HR, 1.41; 95% CIs, 0.89–2.24; *p* = 0.15), indicating that the association between testing modality and OS did not differ between *EGFR*-mutant and *EGFR*-wild-type subgroups (Table 2B).

**Table 2:** Cox proportional hazards analyses of OS: (A) multivariable models and (B) models including an interaction term between testing type and *EGFR* mutation status

A.		
Items	OS (Multivariate analysis)	
	HR (95% CIs)	*p* value
Male	0.96 (0.76–1.25)	0.93
Age, years ≥75	1.61 (1.31–1.98)	**<0.001**
Recurrence	0.88 (0.65–1.20)	0.42
ECOG-PS = 0/1^a^	0.52 (0.37–0.71)	**<0.001**
Adenocarcinoma	0.77 (0.58–1.01)	0.06
Brain metastasis	1.22 (0.96–1.55)	0.10
Liver metastasis	1.61 (1.20–2.14)	**<0.001**
Bone metastasis	1.55 (1.34–2.05)	**<0.001**
PD-L1 ≥50%^b^	0.91 (0.73–1.12)	0.36
*EGFR* mutations detected	0.44 (0.34–0.57)	**<0.001**
Single-plex testing	0.74 (0.59–0.93)	**0.04**

B.		
Items	OS (Cox proportional hazards model including interaction term)	
	HR (95% CIs)	*p*-value
*EGFR* mutation^c^	0.47 (0.34–0.65)	**<0.001**
Type of testing^d^	0.75 (0.57–0.99)	**0.04**
Type of testing × *EGFR* mutations	1.41 (0.89–2.24)	0.15

OS: overall survival, *EGFR*: epidermal growth factor receptor, ECOG-PS: eastern cooperative oncology group performance status, PD-L1: programmed death-ligand 1, CIs: confidence intervals. Bold values indicate statistically significant differences (*p* < 0.05).

a ECOG-PS 0/1 versus ECOG-PS = 2–4 or missing.

b PD-L1 TPS ≥50% versus PD-L1 TPS <49% or unknown.

c Positive vs. negative.

d Single-plex vs. multiplex testing.

### Impact of the number of driver oncogene evaluations and annual progression on clinical outcomes

**Figure 4:**
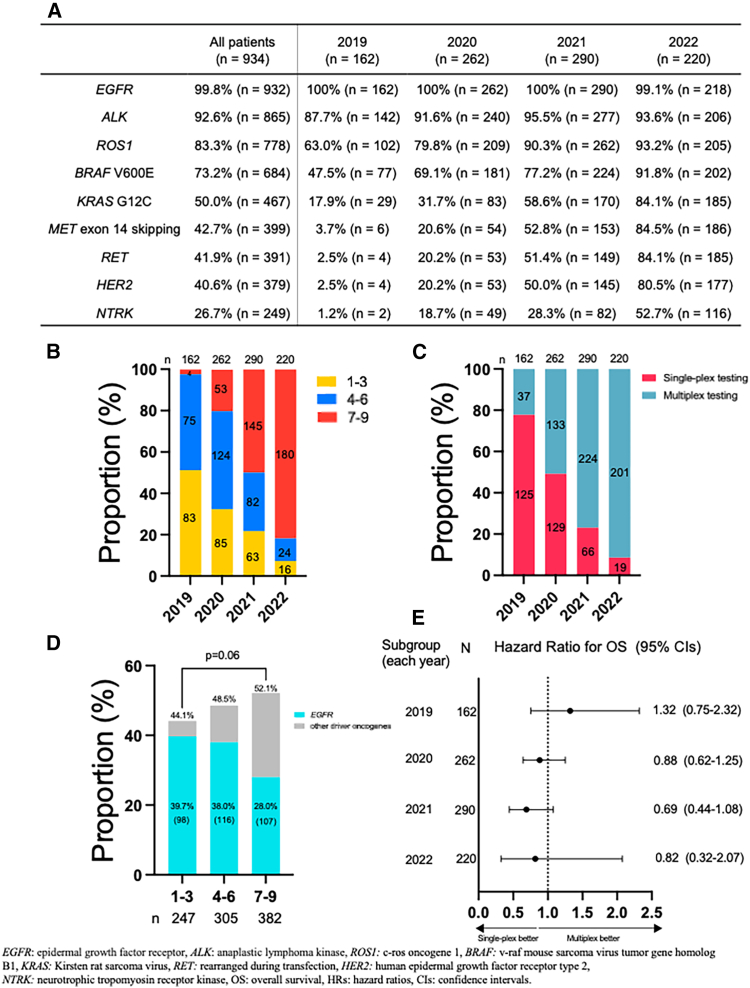
Temporal trends in biomarker testing and driver oncogene evaluation (A–E) Proportion of (A) each driver oncogene evaluated, (B) transition in the number of driver oncogenes evaluated, (C) type of biomarker testing per year, and (D) EGFR and other driver oncogenes detected by the number of driver oncogenes evaluated, and (E) year-stratified hazard ratios for the OS comparing single-plex and multiplex testing, shown as a forest plot. The values shown in the “N” column indicate the number of patients in each subgroup.

The number of driver oncogenes examined by calendar year is depicted in Figure 4A. Overall, *EGFR* mutation was the most evaluated driver oncogene (99.8%, *n* = 932), followed by *ALK* fusions (92.6%, *n* = 865). The proportions of patients evaluated for *ROS1* fusions and *BRAF* V600E mutations were 83.3% (*n* = 778) and 73.2% (*n* = 684), respectively. *KRAS* G12C mutations, *MET* exon 14 skipping mutations, *RET* fusions, *HER2* mutations, and *NTRK* fusions were evaluated in 50.0% (*n* = 467), 42.7% (*n* = 399), 41.9% (*n* = 391), 40.6% (*n* = 379), and 26.7% (*n* = 249) of patients, respectively. In 2022, eight driver oncogenes, excluding *NTRK* fusions, were evaluated in >80% of patients. To assess changes in testing comprehensiveness, the evaluated driver oncogenes were categorized into three groups: 1–3, 4–6, and 7–9. The number of evaluated driver oncogenes increased annually (Figure 4B). In 2022, 91.4% of patients underwent multiplex testing (Figure 4C). Patients evaluated for 7–9 driver oncogenes tended to have a higher driver oncogene detection rate than those evaluated for 1–3 driver oncogenes (52.1% vs. 44.1%, *p* = 0.06). However, the detection rate of *EGFR* mutations was significantly lower (28.0% vs. 39.7%, *p* = 0.003) (Figure 4D). To address potential temporal confounding, we conducted year-stratified survival analyses comparing OS between the single-plex and multiplex testing groups by each year. The year-specific HRs generally favored single-plex testing from 2020 to 2022, although the 95% CIs overlapped unity in each year (Figure 4E).

## Discussion

International guidelines strongly recommend the administration of a molecularly targeted therapy when a driver oncogene is detected in patients with advanced NSCLC. Therefore, comprehensive evaluation of driver oncogenes at the time of diagnosis is crucial.^22^^,^^23^ Recently, multiplex testing has emerged as a viable approach for the simultaneous detection of multiple driver oncogenes in patients with advanced NSCLC in the United States, Europe, Japan, and several other countries.^24^^,^^25^^,^^26^^,^^27^^,^^28^ Nevertheless, multiplex testing has yet to be routinely implemented in many regions, and reimbursement is often limited to single-plex testing for *EGFR* and *ALK* abnormalities.^8^^,^^29^ The ability of multiplex testing simultaneously to evaluate and detect multiple driver oncogenes prevents the diagnostic delay caused by sequential testing associated with single-plex testing. Thus, multiplex diagnostic testing is anticipated to become widely adopted in the clinical management of NSCLC. However, few reports have demonstrated that multiplex testing results in superior clinical outcomes when compared with single-plex testing in patients with NSCLC.^20^^,^^21^ Whether multiplex testing confers clinical benefit in patients with advanced NSCLC remains unclear. To address this clinically important question, we conducted a comprehensive real-world analysis.

To the best of our knowledge, this study is the first to demonstrate an association between biomarker testing modality and clinical outcomes in patients with advanced non-squamous NSCLC. Although patients who underwent single-plex testing exhibited a significantly longer OS than those who underwent multiplex testing did, this finding should be interpreted as an association rather than a causal relationship. No significant difference was observed between the two groups in the overall proportion of patients who received molecularly targeted therapy. However, the detection rate of *EGFR* mutations and the proportion of patients treated with EGFR-TKIs were higher in the single-plex testing group. Although these differences did not reach statistical significance after PSM, the same directional trend favoring single-plex testing remained. Importantly, our data did not fully support the hypothesis that single-plex testing improves OS through increasing *EGFR* mutation detection and subsequent EGFR-TKI use. The interaction between testing modality and *EGFR* mutation status was not statistically significant, suggesting that *EGFR* status did not modify the effect of testing modality in this dataset, although limited statistical power cannot be excluded. In addition, OS did not differ by testing modality among patients who received EGFR-TKIs. Taken together, these findings suggest that the OS difference was not observed within the EGFR-TKI-treated subgroup and may have been largely driven by patients who did not receive EGFR-TKIs. Therefore, we should consider alternative explanations for the observed association. Residual confounding may have contributed to the observed association despite having implemented multivariable adjustment and PSM because testing modality was not randomized and likely reflected institutional practice patterns and clinical decision-making. Unmeasured treatment-related factors, such as subsequent-line therapies, treatment timing and sequencing, and supportive care may also have differed between groups. In addition, patient selection based on clinical factors, including ECOG-PS, could have influenced *EGFR* mutation detection, treatment selection, and clinical outcomes. One plausible explanation for the disparity in the proportion of *EGFR* mutations detected across biomarker testing modalities is inter-platform differences between platforms in analytical sensitivity, including the limits of detection (LOD), and in the spectrum of detectable variants. Single-plex assays may have a lower LOD than that of certain multiplex assays, which may increase the likelihood of false-negative results with multiplex testing, particularly in specimens with low tumor content or low variant allele frequency (AF).^30^^,^^31^^,^^32^ However, this LOD-based explanation remains a hypothesis and cannot be directly verified without specimen-level data, such as tumor cellularity and nucleic acid quality. In addition, pre-analytical factors such as fixation-related nucleic acid integrity and input nucleic acid quantity, as well as analytical factors such as variant-calling and filtering processes and the handling of complex variants, may influence *EGFR* detectability across platforms. As specimen-level quality metrics and raw sequencing data were not uniformly available, we could not directly quantify the contribution of these factors. To provide a quantitative sense of the potential extent of missed *EGFR*-positive cases, we used the reported *EGFR* mutation prevalence in Japan, which is approximately 45%.^17^ In our cohort, the observed *EGFR* detection rates were 39.8% in the single-plex group and 31.4% in the multiplex group, corresponding to an absolute gap of 5.2% and 13.6% from the reference prevalence, respectively. Assuming that a similar underlying prevalence exists in our cohort, these differences suggest that a proportion of *EGFR*-positive cases may have remained unrecognized in each group. This calculation is not a true false-negative rate but rather an illustrative estimate intended to contextualize the potential scale of the impact. The actual *EGFR* prevalence in our cohort may differ from national estimates owing to differences in patient characteristics, specimen-related factors, and institutional practice patterns.

The prognostic association between biomarker testing modality and OS has not been uniformly observed across all clinical contexts. In exploratory subgroup analyses, the survival advantage associated with single-plex testing appeared to be confined to specific clinical strata, whereas no significant differences were observed in other subgroups. These analyses, including the year-stratified survival analyses, were exploratory and may have been affected by limited sample sizes and event counts within individual strata and calendar years, leading to statistical variability. Consequently, the subgroup- and year-specific estimates were less precise, with wide 95% CIs, and the absence of statistical significance within individual strata or years should not be interpreted as evidence of no difference among them. For similar reasons, comparisons by EGFR-TKI generation within the EGFR-TKI subgroup may be underpowered, particularly in the multiplex group, because few patients received first-generation EGFR-TKIs and should thus be interpreted cautiously. In contrast, multiplex testing identified a higher proportion of actionable driver alterations, excluding *EGFR* and *ALK*, than single-plex testing did. However, only 6.6% of patients in the multiplex cohort ultimately received matched targeted therapies. One explanation for this discrepancy is the timing of drug approvals during the study period. For instance, in Japan, sotorasib for *KRAS* G12C-mutant NSCLC was approved for insurance coverage in January 2022; trastuzumab deruxtecan for *HER2*-mutant NSCLC was approved in August 2023; and insurance coverage for both *KRAS*- and *HER2*-targeted therapies is currently restricted to second-line or later treatment settings.

Although multiplex testing was associated with a slightly longer median TAT than that of single-plex testing, this difference was clinically modest despite the statistical significance measured. As defined in the Supplementary Materials and Methods, TAT refers to the time from specimen submission to documentation of the test result in the medical record. The weak correlation between TAT and the interval from diagnosis to treatment initiation suggests that assay processing time is unlikely to meaningfully delay treatment or be a major driver of the observed OS difference. Therefore, the small difference in TAT should be cautiously interpreted when considering clinical outcomes. Consistent with this interpretation, empirical systemic therapy was initiated before biomarker confirmation more frequently in the multiplex testing group than in the single-plex testing group. However, this observation does not establish a causal role for TAT in the OS difference observed and may also reflect institutional practice and residual confounding.

Our findings should not be interpreted as evidence that limited single-plex testing is superior to multiplex approaches. International guidelines uniformly recommend multiplex testing strategies to ensure that all actionable oncogenic drivers are assessed at diagnosis, and our study does not contradict this principle. Rather, our results highlight specific limitations of currently available multiplex assays in Japan, particularly with respect to *EGFR* detection sensitivity. For *ALK* fusion, both the single-plex and multiplex testing exhibited a high concordance rate of over 98.0%.^8^^,^^33^ Similar to previous reports, our study indicated that the detection rate of *ALK* fusion was not influenced by the test of type used.

Among patients with non-squamous NSCLC who received ICI-containing regimens as first-line therapy, those who underwent single-plex testing demonstrated a longer OS than those who underwent multiplex testing did. A previous study showed that patients with advanced NSCLC harboring *EGFR* mutations derive limited benefit from ICIs.^34^ In this context, a higher proportion of undetected *EGFR* mutations in the multiplex testing group may have led to the inappropriate administration of immunotherapy-containing regimens and poorer survival outcomes. Moreover, only a small proportion of patients in the multiplex cohort who received ICI-containing regimens were treated with angiogenesis inhibitors (5.4%, 32 of 595), which are known to have potential efficacy in *EGFR*-mutant NSCLC. The limited benefit of immunotherapy with angiogenesis inhibitors in this population may have further contributed to the observed survival differences. Biologically, NSCLC harboring *EGFR* mutations is often associated with lower tumor mutational burden and a less inflamed tumor microenvironment, which are features linked to the reduced responsiveness to ICIs. These clinicobiological characteristics may amplify the adverse impact of unrecognized *EGFR* mutations when ICI-based regimens are selected in clinical practice.^35^

For patients with undetected driver oncogenes following initial testing, further genomic evaluation is warranted. Comprehensive genomic profiling (CGP) assays have been reported to identify additional actionable driver alterations, including *EGFR* mutations and *ALK* fusions, even after receiving negative results from single-plex or multiplex testing.^36^^,^^37^^,^^38^ Accordingly, CGP assays may represent an important complementary approach regardless of the initial testing modality. In particular, a sequential strategy consisting of single-plex *EGFR* testing followed by CGP for patients who remain negative on initial testing may represent a practical approach in settings where *EGFR* mutations are highly prevalent. In the future, improvements in multiplex assay design, such as lowering the LOD and transitioning from amplicon-based to hybrid-capture sequencing, may help overcome the current sensitivity limitations, particularly for *EGFR* mutations. Notably, newer multiplex platforms with improved analytical sensitivity, achieving an *EGFR* LOD ≤1%, have been introduced in Japan. For example, the lung cancer compact panel has reported an *EGFR* LOD of ≤1%, suggesting its potential to reduce false-negative *EGFR* results while maintaining broad driver coverage.^39^ This next generation assay represents a practical direction for improving the sensitivity of multiplex testing. Nevertheless, continued validation in real-world settings will be essential because analytical performance may vary according to specimen quality and pre-analytical factors. Although the present study was conducted in Japan, the findings may also be relevant to other Asian countries where *EGFR* mutation prevalence is similarly high, such as China and South Korea.^40^^,^^41^^,^^42^ However, generalizability may vary depending on differences in multiplex assay platforms, sequencing technologies, and the timing of approval and reimbursement for targeted therapies beyond *EGFR* mutations. Validation in additional real-world Asian cohorts is warranted.

### Limitations of the study

This study had several limitations. First, we were unable to obtain detailed specimen-level information from medical records, including tumor cellularity and nucleic acid yield, which may have affected the detection of driver oncogenes. Samples with low tumor cellularity are more susceptible to false-negative results when analyzed using assays with higher LOD, which may have contributed to the observed differences in *EGFR* mutation detection rates and clinical outcomes. However, no significant differences were observed between the single-plex and multiplex testing groups regarding the proportions of tissue versus cytology specimens or primary versus metastatic lesion samples. In addition, most baseline patient characteristics were comparable between the two groups, except for ECOG-PS. Second, because raw sequencing data were not available for centralized review in this retrospective multicenter study, *EGFR* mutations were ascertained from routine clinical reports generated via commercial bioinformatic workflows. Therefore, we could not perform centralized manual review in the integrative genomics viewer using binary alignment map files to assess whether complex *EGFR* alterations, including complex deletions, might have been missed by the commercial workflows. Third, this study could not directly determine the proportion of false-negative results associated with each biomarker testing modality. Confirmation of this hypothesis would require head-to-head comparisons of single-plex and multiplex testing performed on the same specimens under identical experimental conditions. Fourth, this study was retrospective and lacked randomization, and the selection of single-plex or multiplex testing was not standardized across participating institutions. These factors may have introduced selection bias and confounding, which could partly explain the observed differences in *EGFR* mutation detection rates and clinical outcomes. Although PSM was performed to reduce baseline imbalances, residual confounding from unmeasured factors may have remained. These factors include institutional practice patterns, physician treatment preferences, and unrecorded variations in pre-analytical processes. In addition, gender-related information was not available in this retrospective dataset, and the potential influence of gender on the study results could not be evaluated. Therefore, the findings should be interpreted as associative rather than causal. Fifth, detailed differences in post-biomarker treatment strategies, including supportive care and treatment adherence, were not fully evaluated and may have influenced the OS. In addition, the timing of imaging to assess treatment efficacy was not predefined. However, treatment response was evaluated by the physician in charge at each institution, following RECIST version 1.1. The possibility of immortal-time bias cannot be completely disregarded because treatment initiation and follow-up schedules were not strictly standardized in this retrospective design. In addition, the study period partially overlapped with the COVID-19 pandemic, which may have caused minor logistical delays in specimen processing and treatment initiation.

In conclusion, in this Japanese cohort, single-plex testing was associated with higher *EGFR* mutation detection rates and a longer OS than those of currently available multiplex assays, such as the Oncomine Dx target test and the Amoy 9-in-1 panel. These findings should be interpreted as an association rather than a causal relationship, and may, in part, reflect platform-dependent limitations in multiplex assay performance. Comprehensive multiplex testing is still recommended by major international guidelines for patients with advanced NSCLC.^24^^,^^25^^,^^26^^,^^27^ As multiplex technologies continue to evolve, improvements in test performance may mitigate the current advantage of single-plex testing. Future efforts should focus on optimizing multiplex assay sensitivity and diagnostic strategies to ensure the timely and accurate identification of actionable driver oncogenes.

## Resource availability

### Lead contact

Requests for further information and resources should be directed to and will be fulfilled by the lead contact, Tadaaki Yamada (tayamada@koto.kpu-m.ac.jp).

### Materials availability

This study did not generate new unique reagents.

### Data and code availability

#### Data

All data reported in this paper will be shared by the lead contact upon request.

#### Code

This paper does not report original code.

#### Additional information

Any additional information required to reanalyze the data reported in this paper is available from the lead contact upon request.

## Acknowledgments

We thank the patients, their families, and all investigators involved in this study. Additionally, we thank Editage (www.editage.jp) for their help with English language editing.

## Author contributions

Conceptualization, methodology, formal analysis, investigation, writing—original draft, writing—review and editing, and visualization, M.I.; conceptualization, methodology, formal analysis, investigation, writing—original draft, writing—review and editing, project administration, visualization, and supervision, T.Y.; investigation and writing—review and Editing, Y.G.; investigation and writing—review and editing, T.O.; investigation and writing—review and editing, H.T.; investigation and writing—review and editing, T.H.; investigation and writing—review and editing, A.Y.; investigation and writing—review and editing, S.S.; investigation and writing—review and editing, A.O.; investigation and writing—review and editing, K.J.; investigation and writing—review and editing, H.K.; investigation and writing—review and editing, N.I.; investigation and writing—review and editing, M.F.; investigation and writing—review and Editing, T.Y.; investigation and writing—review and editing, T.H.; investigation and writing—review and editing, H.K.; investigation and writing—review and editing, Y.K.; investigation and writing—review and editing, K.M.; investigation and writing—review and editing, M.I.; investigation and writing—review and editing, T.K.; investigation and writing—review and editing, K.T.

## Declaration of interests

T.Y. received grants from Pfizer, Ono Pharmaceutical, Janssen Pharmaceutical, and Takeda Pharmaceutical, as well as personal fees from Eli Lilly.

H.T. received grants in the form of personal fees from AstraZeneca and Chugai Pharmaceutical. K.

H.K. received personal fees from Ono Pharmaceutical Co. Ltd., Bristol-Myers Squibb KK, Chugai Pharmaceutical Co. Ltd., AstraZeneca KK, Taiho Pharmaceutical Co. Ltd., Eli Lilly Japan KK, and MSD KK, outside the purview of the submitted work.

K.T. received grants from Chugai Pharmaceutical and Ono Pharmaceutical, as well as personal fees from AstraZeneca, Chugai Pharmaceutical, MSD, Eli Lilly, Boehringer Ingelheim, and Daiichi Sankyo.

## STAR★Methods

### Key resources table

**Table:** 

REAGENT or RESOURCE	SOURCE	IDENTIFIER
**Critical commercial assays**		

cobas EGFR Mutation Test v2	Roche Molecular Systems	https://diagnostics.roche.com/
Oncomine Dx Target Test	Thermo Fisher Scientific	https://www.thermofisher.com/
AmoyDx Pan Lung Cancer PCR Panel	Amoy Diagnostics	https://www.amoydiagnostics.com/

**Software and algorithms**		

EZR version 1.54	Saitama Medical Center Jichi Medical University	https://www.jichi.ac.jp/usr/hema/EZR/statmedEN.html
GraphPad Prism version 8.0	GraphPad Software	https://www.graphpad.com/

### Experimental model and study participant details

The inclusion criteria were patients (1) diagnosed with consecutive advanced non-squamous NSCLC (stage Ⅳ, including postoperative recurrence based on the American Joint Committee on Cancer Staging Manual, version 8) and (2) who received any form of anticancer therapy between June 2019 and December 2022 at 12 institutions in Japan. Patients who did not undergo biomarker testing or whose biomarker testing failed were excluded. Clinical data were retrospectively collected from electronic medical records. The following data were collected: age, sex, ECOG-PS, smoking status, tumor histology, staging, metastatic sites, sample type (tissue or cytology specimens and primary or metastatic lesions), PD-L1 tumor proportion score (TPS) and driver oncogene status, type of biomarker testing (single-plex or multiplex), treatment regimens, and OS. This multicenter retrospective cohort study was conducted in accordance with the Declaration of Helsinki of the World Medical Association.^43^ The study protocol was approved by the Ethics Committee of the Kyoto Prefectural University of Medicine (ERB-C-2982-1), as well as by the ethics review boards of all participating institutions: Fujita Health University School of Medicine (HM23-283), Hyogo Medical University (202401-072), Nagasaki University Graduate School of Biomedical Sciences (23121813-2), Fukuchiyama City Hospital (5-74), Japanese Red Cross Kyoto Daini Hospital (S2023-38), Saiseikai Suita Hospital (2023-70), Japanese Red Cross Kyoto Daiichi Hospital (1662), Omi Medical Center (2021-06), Otsu City Hospital (176), Uji-Tokushukai Medical Center (TGE01607-007), and Matsushita Memorial Hospital (23027). The requirement for obtaining informed consent from the patients was waived because of the retrospective nature of the study. Instead, an opt-out approach was implemented at each institution. Information regarding the study, including the option to decline participation, was made publicly available through institutional websites, allowing patients or their families to notify the study team if they wished to refuse the use of their data. During the study period, no patients or families opted out.

### Method details

Patients were allocated to either the single-plex or multiplex testing groups according to the type of initial biomarker testing performed in routine clinical practice at each participating institution. This allocation was not randomized and reflected real-world clinical decision-making. Based on pivotal studies, driver oncogenes were defined as *EGFR* mutations, *ALK* fusion, *ROS1* fusion, *BRAF* V600E, *MET* exon 14 skipping mutations, *RET* fusion, *KRAS* G12C mutations, *HER2* mutations, and *NTRK* fusion.^3^^,^^4^^,^^5^^,^^6^^,^^7^^,^^44^^,^^45^^,^^46^^,^^47^^,^^48^^,^^49^ PD-L1 TPS in tumor cells was assessed using immunohistochemistry with the PD-L1 22C3 pharmDx antibody (clone 22C3; Dako North America, Inc. Carpinteria, CA, USA). Single-plex testing was defined as a molecular diagnostic assay designed to evaluate a single driver oncogene per assay. Single-plex testing primarily included PCR-based assays, such as the PNA-LNA PCR clamp test and cobas *EGFR* Mutation Test v2 for *EGFR*, as well as fluorescence *in situ* hybridization or PCR-based assays for *ALK* fusions. In contrast, multiplex testing was defined as diagnostic platforms capable of simultaneously evaluating multiple driver oncogenes within a single assay and included both next-generation sequencing-based and multiplex PCR-based methods. In Japan, multiplex testing is primarily performed using the Oncomine Dx Target Test or Amoy 9-in-1 panel. The Oncomine Dx Target Test was first approved in April 2018 as a companion diagnostic for *BRAF* V600E and reimbursed by the national health insurance system in December 2018, with a subsequent expansion in 2019 to include *EGFR*, *ALK*, and *ROS1* alterations. The Amoy 9-in-1 panel has been clinically available since January 2022. The LODs for single-plex assays were a 0.1–1% AF for the PNA-LNA PCR Clamp test and 3–5% AF for the cobas *EGFR* Mutation Test v2. In contrast, the LOD for multiplex assays were approximately a 6–8% AF for *EGFR* mutations with the Oncomine Dx Target Test and approximately 1% AF with the Amoy 9-in-1 panel.^30^^,^^31^^,^^32^ A comprehensive list of reportable gene variants detected by CDx-approved single-plex and multiplex assays is provided in Table S7 (*EGFR*, *KRAS*, *BRAF*, and *ERBB2* variants). The complete list of fusion genes reportable by the Oncomine Dx Target Test and Amoy 9-in-1 panel is summarized in Table S8 (*ALK*, *ROS1*, and *NTRK* variants).^50^ Both multiplex panels employed in this study covered all common *EGFR* exons (18–21), including clinically relevant mutations, such as T790M and exon 20 insertions. Clinical data were collected using a standardized case report form specifically developed for this multicenter study. A uniform data dictionary was applied to ensure data consistency across institutions, and anonymized datasets were centrally compiled at the Kyoto Prefectural University of Medicine. For categorical variables, missing data were treated as a separate category and included in all analyses, including the propensity score model and post-matching comparisons.

### Quantification and statistical analysis

Statistical analyses were performed to compare baseline characteristics, biomarker detection rates, treatment administration rates, and OS between the single-plex and multiplex testing groups. In this study, n represents the number of patients unless otherwise specified. All statistical tests were two-sided, and statistical significance was set at p < 0.05. Age was analyzed using the Wilcoxon rank-sum test. Dichotomous variables were compared using the chi-square or Fisher’s exact test, as appropriate. Survival curves were calculated using the Kaplan–Meier method, and differences compared using the log-rank test. For proportion-based outcomes, such as biomarker detection and treatment administration rates, 95% confidence intervals (CIs) were calculated using the Wilson method. Cox proportional hazards models were used in the univariate and multivariable analyses to estimate hazard ratios and 95% CIs. Multivariable Cox proportional hazards models were used to evaluate the association between the type of testing and OS. The proportional hazards assumption was assessed using Schoenfeld residuals. Based on pivotal reports, the following variables were selected as covariates: sex, age (≥ 75 years), staging, Eastern Cooperative Oncology Group performance status (ECOG-PS), histology, presence of brain, bone, or liver metastasis, PD-L1 tumor proportion score (TPS) ≥ 50%, epidermal growth factor receptor (*EGFR*) mutation status, and driver oncogene evaluation using single-plex testing.^9^^,^^51^^,^^52^^,^^53^^,^^54^^,^^55^^,^^56^^,^^57^^,^^58^^,^^59^ To address potential institutional selection bias, a sensitivity analysis was performed using a multivariable Cox model that included the institution as an additional categorical covariate. To further evaluate whether the prognostic effect of testing type was modified by *EGFR* mutation status, we fitted an additional Cox regression model that included an interaction term between testing type and *EGFR* mutation status. To minimize potential selection bias between patients who underwent single-plex and multiplex testing, a propensity score matching analysis was performed. The propensity score was estimated using a logistic regression model that included age, sex, ECOG-PS, stage, smoking status, and PD-L1 TPS. Nearest-neighbor matching was conducted at a 1:1 ratio without replacement, with a caliper of 0.2 of the standard deviation of the logit of the propensity score. To evaluate the association between TAT and the time to treatment initiation, we assessed the correlation between TAT and the interval from diagnosis to first-line therapy using Spearman’s rank correlation analysis. Results were visualized using scatter plots with fitted regression lines. All statistical analyses were performed using EZR (v.1.54; Saitama Medical Center, Jichi Medical University, Saitama, Japan)^60^ and Prism (v.8.0; GraphPad Software, San Diego, CA, USA). Additional methodological details regarding specimen collection, testing workflow, and efficacy analysis are provided in Methods S1.
