# Supplementary material for: Initial biomarker testing strategies and clinical outcomes in advanced non-small cell lung cancer
Source: iScience. 2026 May 15;29(6):115966. doi: 10.1016/j.isci.2026.115966 (PMC13206649; doi:10.1016/j.isci.2026.115966)
Supplement: Document S1. Figures S1–S12, Tables S1–S8, and Methods S1 [file mmc1.pdf]

iScience, Volume *29*

Supplemental information

Initial biomarker testing strategies and clinical outcomes in advanced non-small cell lung cancer

Masaki Ishida, Tadaaki Yamada, Yasuhiro Goto, Taiichiro Otsuki, Hirokazu Taniguchi, Taishi Harada, Akihiro Yoshimura, Shinsuke Shiotsu, Asuka Okada, Kazuki Jinno, Hibiki Kanda, Noeru Inoguchi, Mototaka Fukui, Takahiro Yamada, Tae Hata, Hayato Kawachi, Yuki Katayama, Kenji Morimoto, Masahiro Iwasaku, Takashi Kijima, and Koichi Takayama

Methods S1

Detailed description of specimen collection and testing workflow, and efficacy analysis

Specimen collection and testing workflow

Tumor specimens for biomarker testing were obtained from tissue or cytology samples routinely collected at each participating institution. Nucleic acid extraction and quality control procedures were performed according to the manufacturer's instructions for each assay. The same manufacturer-specified reagents and quality-control criteria were applied across centers that performed the same assay. All molecular testing was conducted in ISO 15189–accredited (or equivalent Ministry of Health, Labour and Welfare–approved) clinical diagnostic laboratories. Licensed clinical laboratory technologists performed all assays according to standardized operating procedures. Single-plex testing was generally performed at in-hospital or external commercial laboratories, whereas multiplex testing was typically centralized at specific reference laboratories depending on the platform used. During the study period, no major center-specific modifications were introduced that would differentially affect single-plex versus multiplex testing. Turnaround time (TAT) was defined as the number of days from specimen submission for biomarker testing to documentation of the test result in the medical records. In routine clinical practice across the participating institutions, patients with suspected non-small cell lung cancer (NSCLC) initially underwent imaging evaluation at first. This was followed by tissue acquisition via bronchoscopy, percutaneous needle biopsy, thoracentesis, or surgical resection if malignancy was suspected. Following the pathological confirmation of NSCLC, biomarker testing was

performed to assess driver oncogene alterations using single-plex or multiplex testing platforms with programmed death-ligand 1 (PD-L1) immunohistochemistry. The biomarker results were subsequently used to guide the selection of first-line treatment. A schematic representation of the diagnostic-to-treatment workflow is shown in **Figure S12**.

Efficacy analysis

Overall survival (OS) was defined as the time from the first administration of any treatment to death from any cause. The data cutoff was set to July 31, 2023. The median (IQR) follow-up time for the censored cases was 20.7 (11.4–49.4) months.

Figure S1. Correlation between turnaround time for biomarker testing and the time from diagnosis to initiation of first-line therapy.

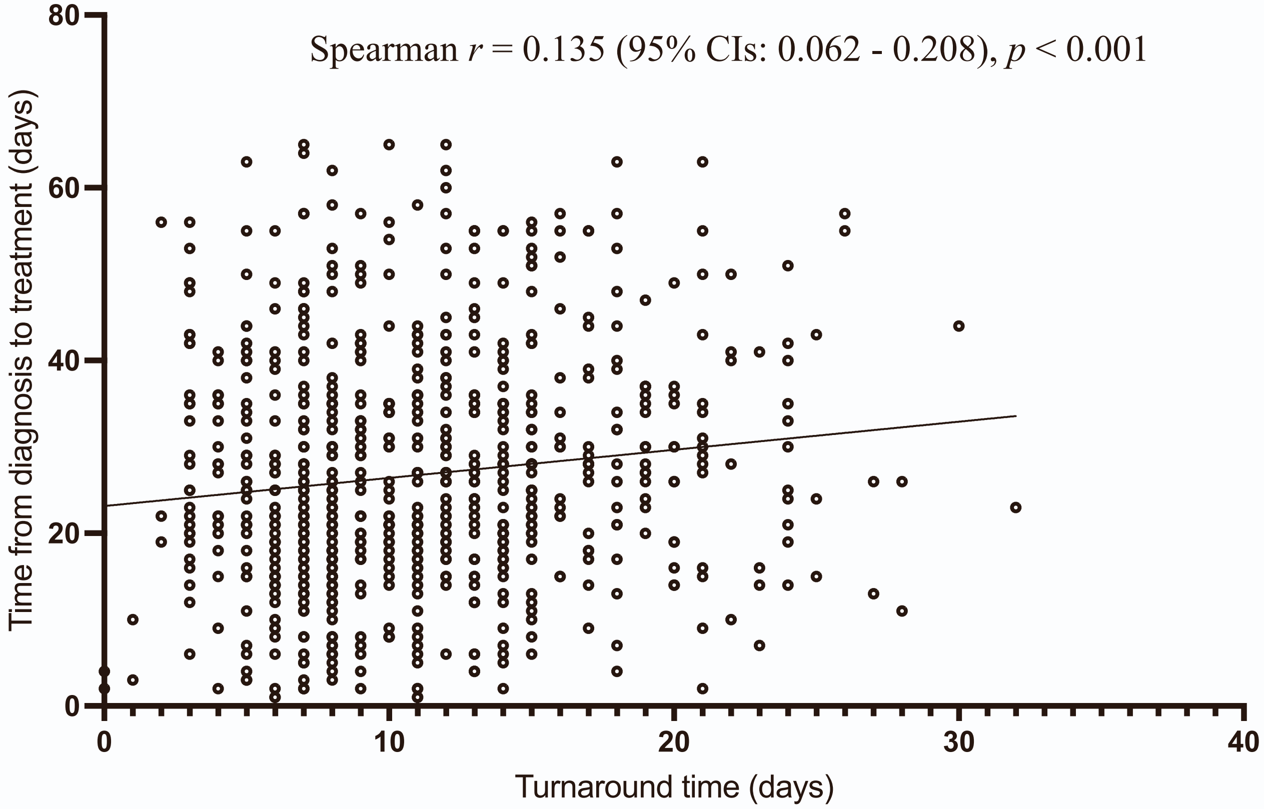

CIs: confidence intervals.

Figure S2. Proportion of (A) patients who underwent single-plex testing (n = 339), and (B) patients who underwent multiplex testing (n = 595).

A.

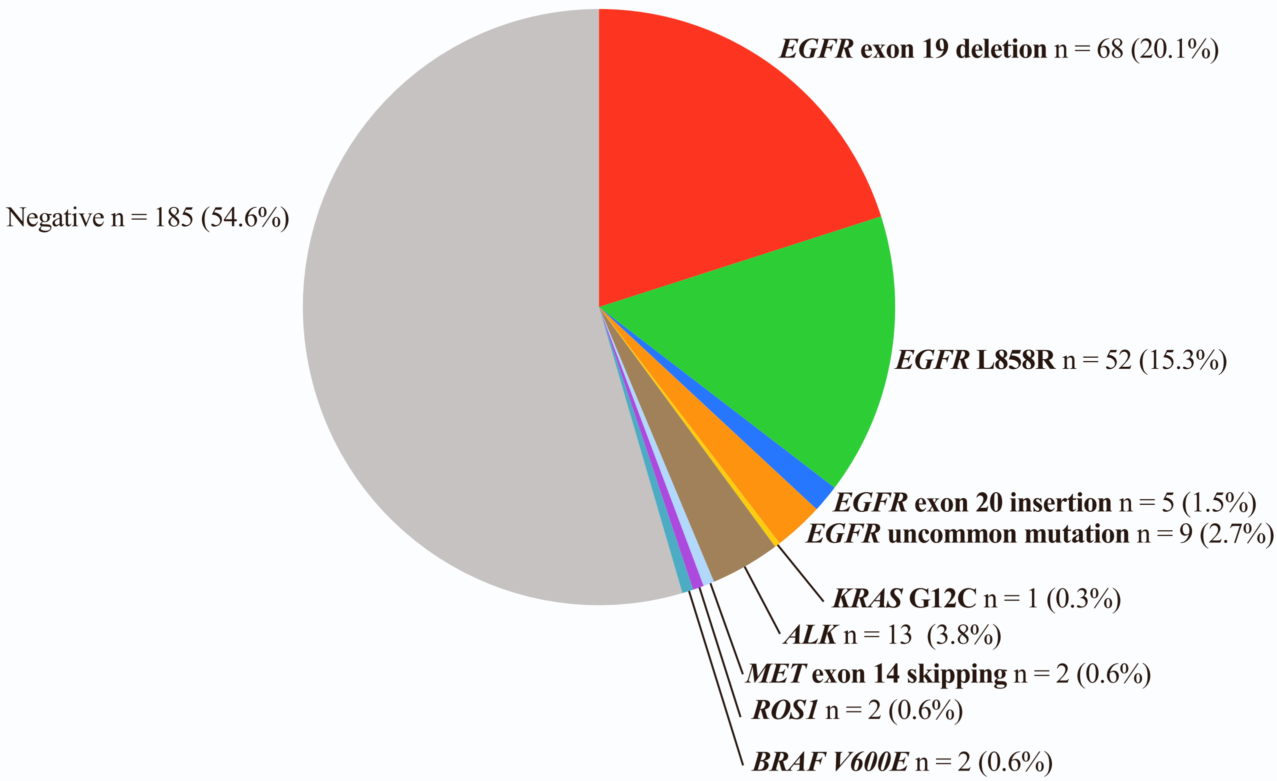

B.

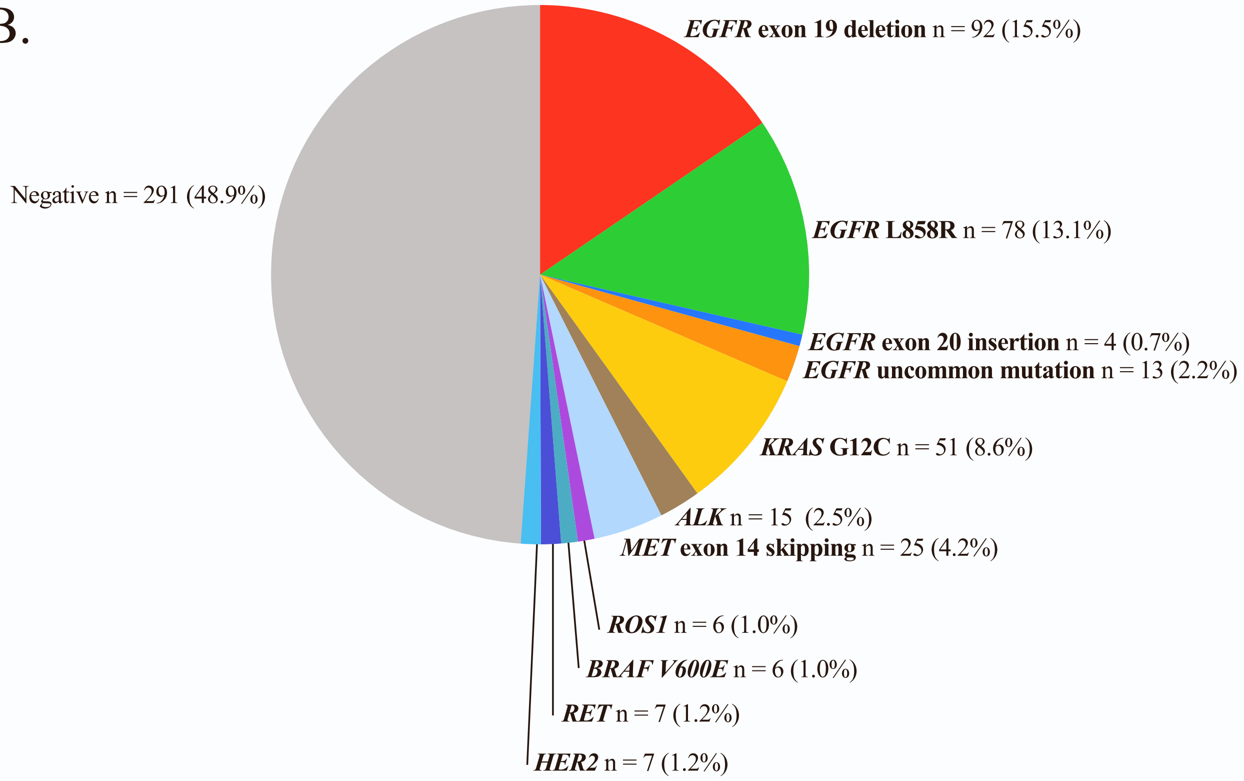

EGFR: epidermal growth factor receptor, *ALK*: anaplastic lymphoma kinase, *ROS1:* c-ros oncogene 1, *BRAF:* v-raf mouse sarcoma virus tumor gene homolog B1, *KRAS:* Kirsten rat sarcoma virus, *RET:* rearranged during transfection, *HER2:* human epidermal growth factor receptor type2.

Figure S3. Distribution of *EGFR* mutation positivity (A) across 12 institutions and (B) comparison of *EGFR* mutation subtypes according to each testing platform.

A.

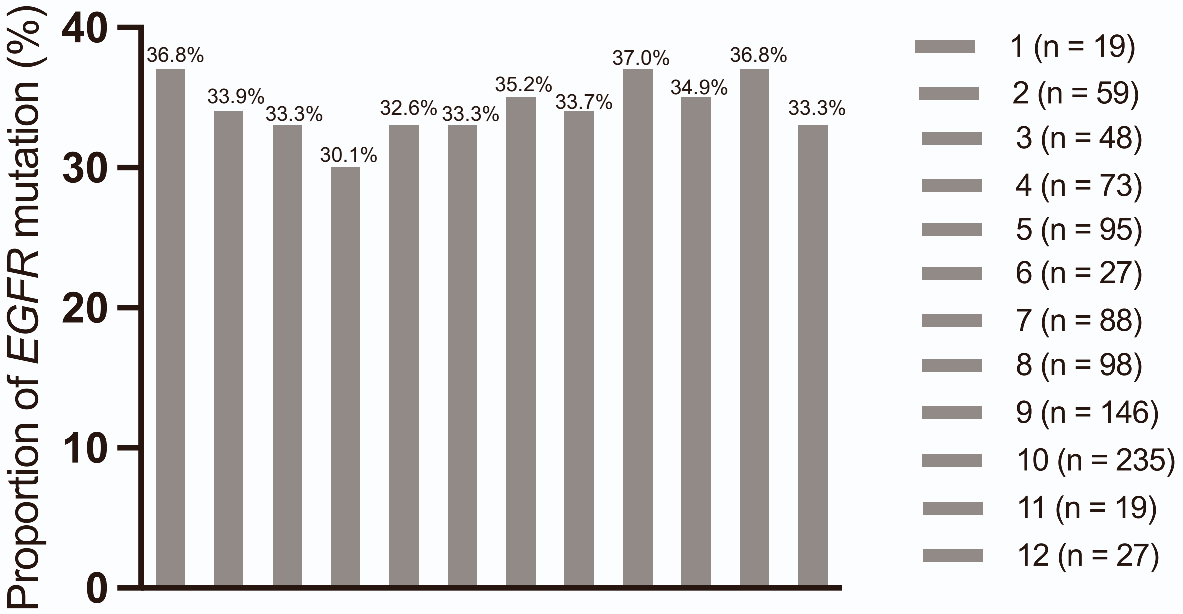

B.

Items	Single-plex (n = 339)		Multiplex (n = 595)			
	Cobas EGFR Mutation Test v2 (n = 323)	PNA-LNA PCR clamp (n = 16)	Oncomine Dx Target Test (n = 434)	i-densy (n = 77)	Amoy 9-in-1 panel (n = 63)	LC-SCRUM Japan program (n = 21)
EGFR mutations	126 (39.0%)	8 (50.0%)	135 (31.3%)	35 (45.5%)	13 (20.6%)	4 (19.0%)
subtype						
Exon 19 deletion	64 (19.8%)	4 (25.0%)	69 (15.9 %)	16 (20.8%)	5 (7.9%)	2 (9.5%)
L858R	50 (15.5%)	2 (12.5%)	53 (12.2%)	18 (23.4%)	6 (9.5%)	1 (4.8%)
Exon 20 insertion	4 (1.1%)	1 (6.3%)	3 (0.7%)	-	1 (1.6%)	-
Uncommon mutations	8 (2.5%)	1 (6.3%)	10 (2.3%)	1 (1.3%)	1 (1.6%)	1 (4.8%)

EGFR: epidermal growth factor receptor.

Figure S4. Annual trends in (A) *EGFR* mutation detection rates and (B) osimertinib utilization according to the type of biomarker testing.

A.

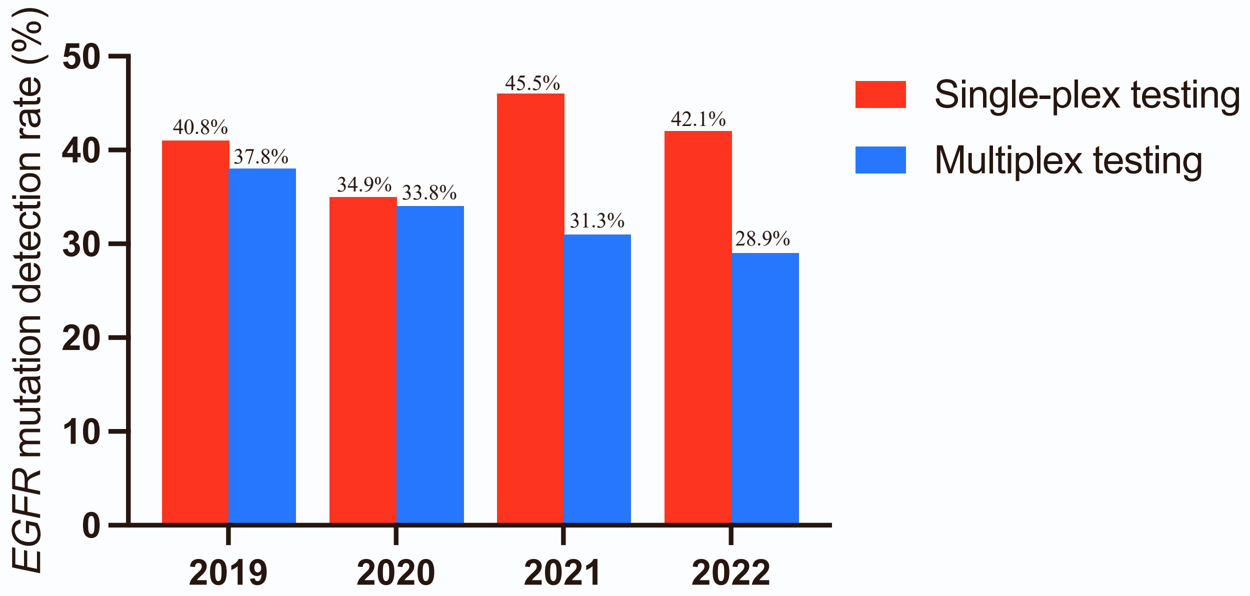

B.

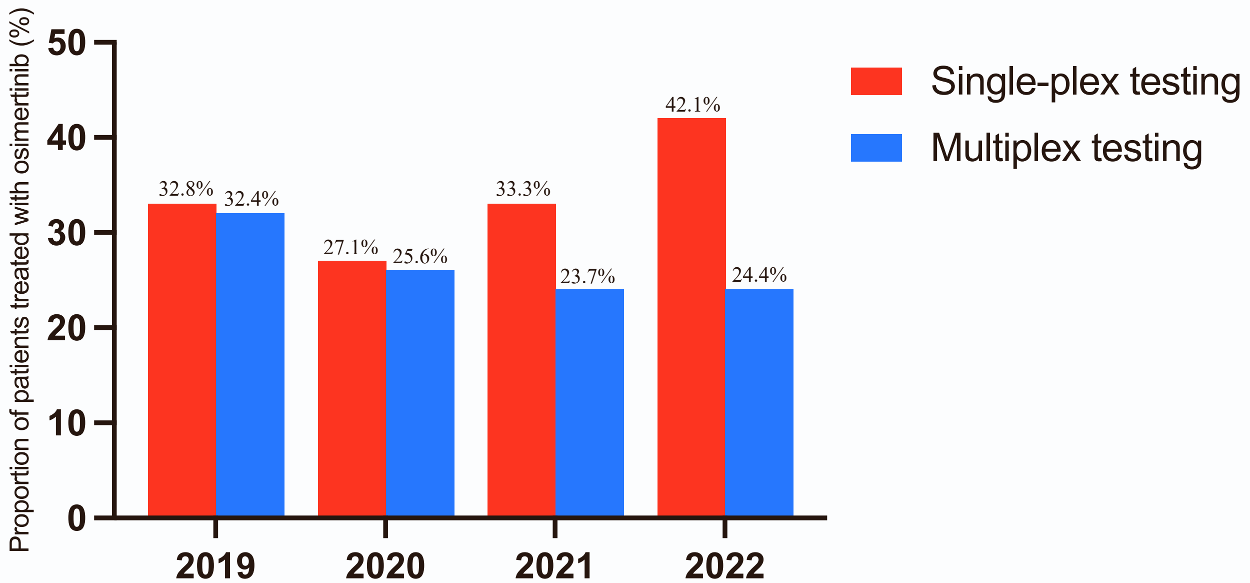

EGFR: epidermal growth factor receptor.

Figure S5. Proportion of (A) driver oncogenes, (B) patients who received molecular targeted therapy according to the type of biomarker testing, and (C) proportion of matched targeted therapy by each driver alteration, all excluding *EGFR* and *ALK* abnormalities.

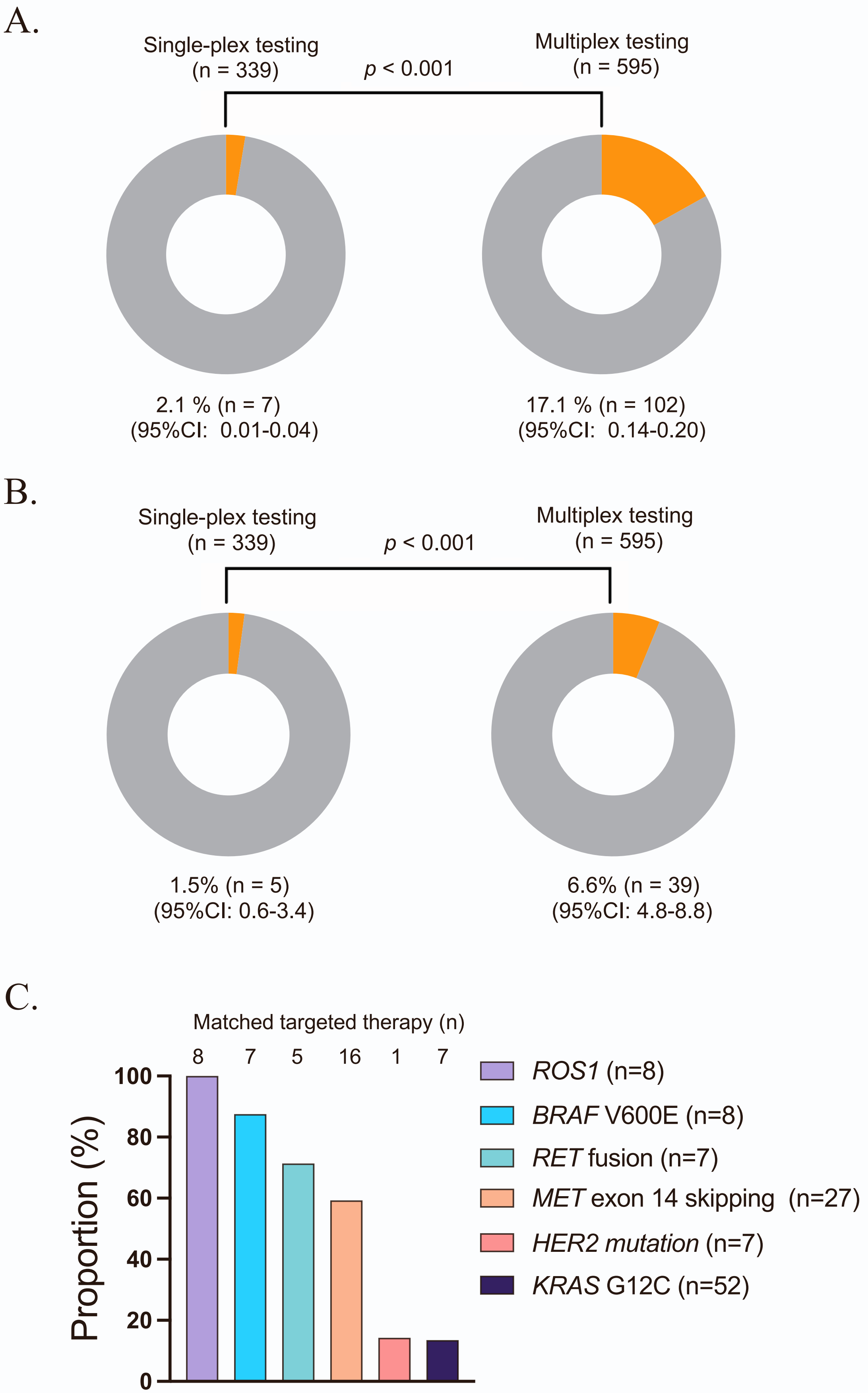

EGFR: epidermal growth factor receptor, *ALK*: anaplastic lymphoma kinase, *ROS1:* c-ros oncogene 1, *BRAF:* v-raf mouse sarcoma virus tumor gene homolog B1, *KRAS:* kirsten rat sarcoma virus, *RET:* rearranged during transfection, *HER2:* human epidermal growth factor receptor type2, CI: confidence interval.

Figure S6. Proportion of (A) *EGFR* mutation positivity, (B) patients who received *EGFR*-TKIs, and (C) Kaplan–Meier curves for OS of patients with advanced non-squamous NSCLC, after propensity score matching according to biomarker testing modality. Median OS values with 95% CIs are shown in the plot.

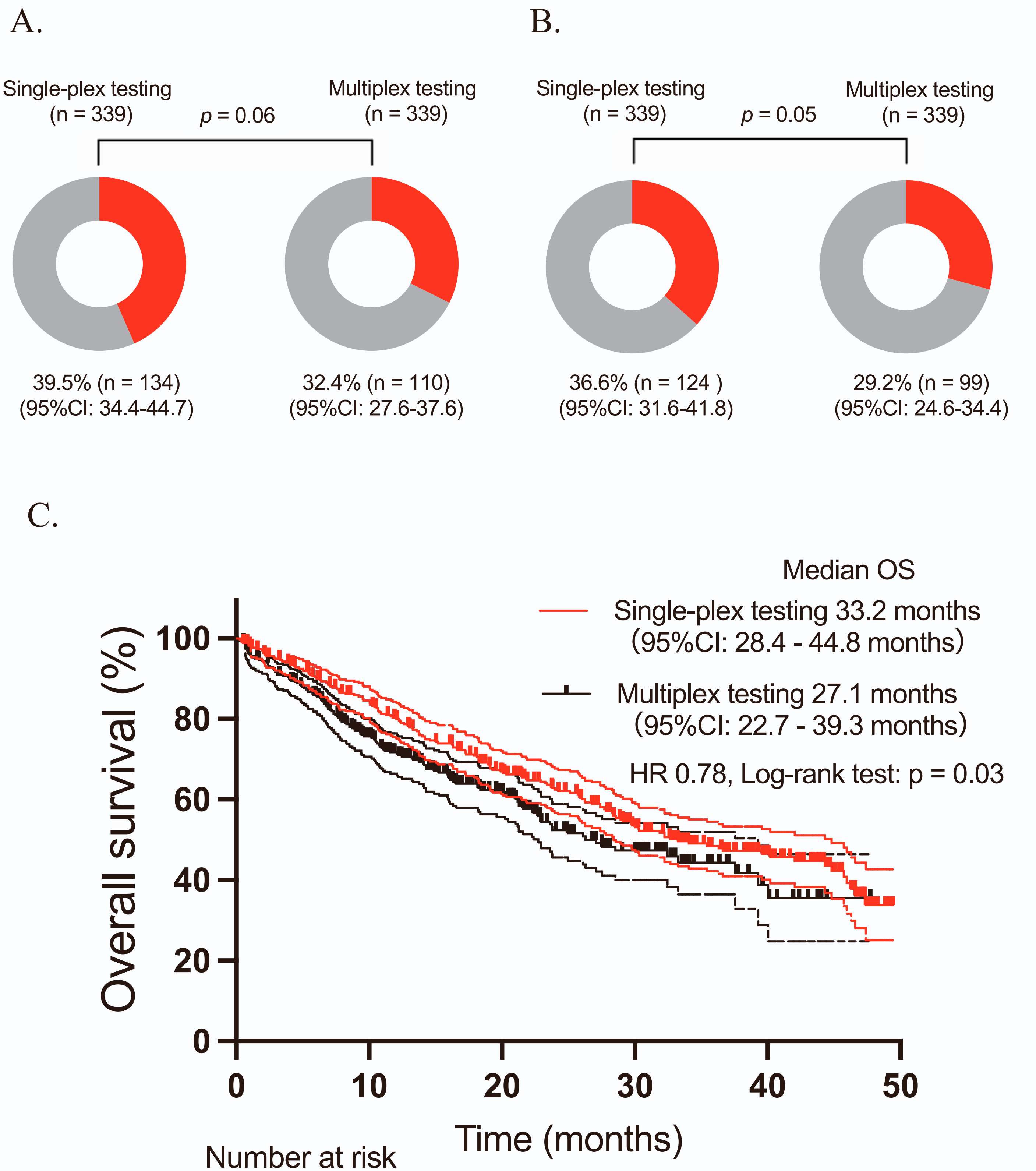

Number at risk

Single-plex	339	261	187	115	59	0
Multiplex	339	217	113	47	12	0

EGFR: epidermal growth factor receptor, TKIs: tyrosine kinase inhibitors, OS: overall survival, NSCLC: non-small cell lung cancer, CIs: confidence intervals.

Figure S7. Kaplan–Meier curves OS of all patients with advanced non-squamous NSCLC.

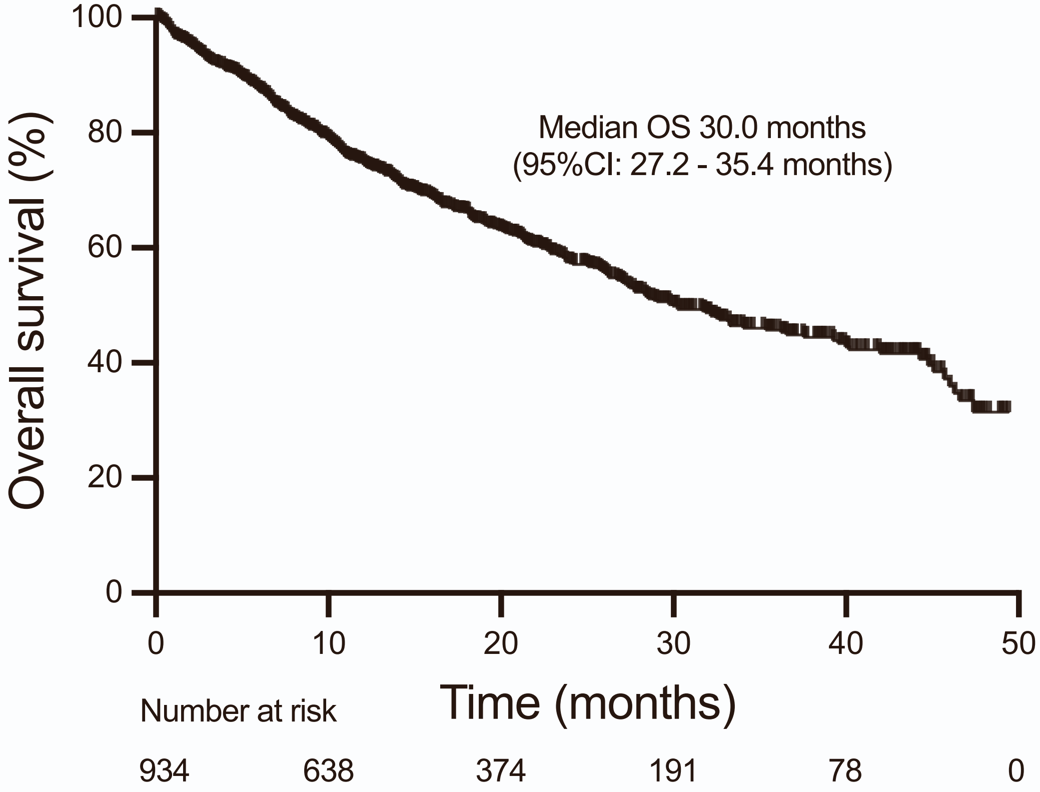

OS: overall survival, NSCLC: non-small cell lung cancer, CI: confidence interval.

Figure S8. Forest plot for OS according to biomarker testing modality in subgroups defined by ECOG-PS, Stage, and PD-L1 status. HRs and 95% CIs were estimated using univariate Cox proportional hazards models. The values shown in the "N" column indicate the number of patients in each subgroup.

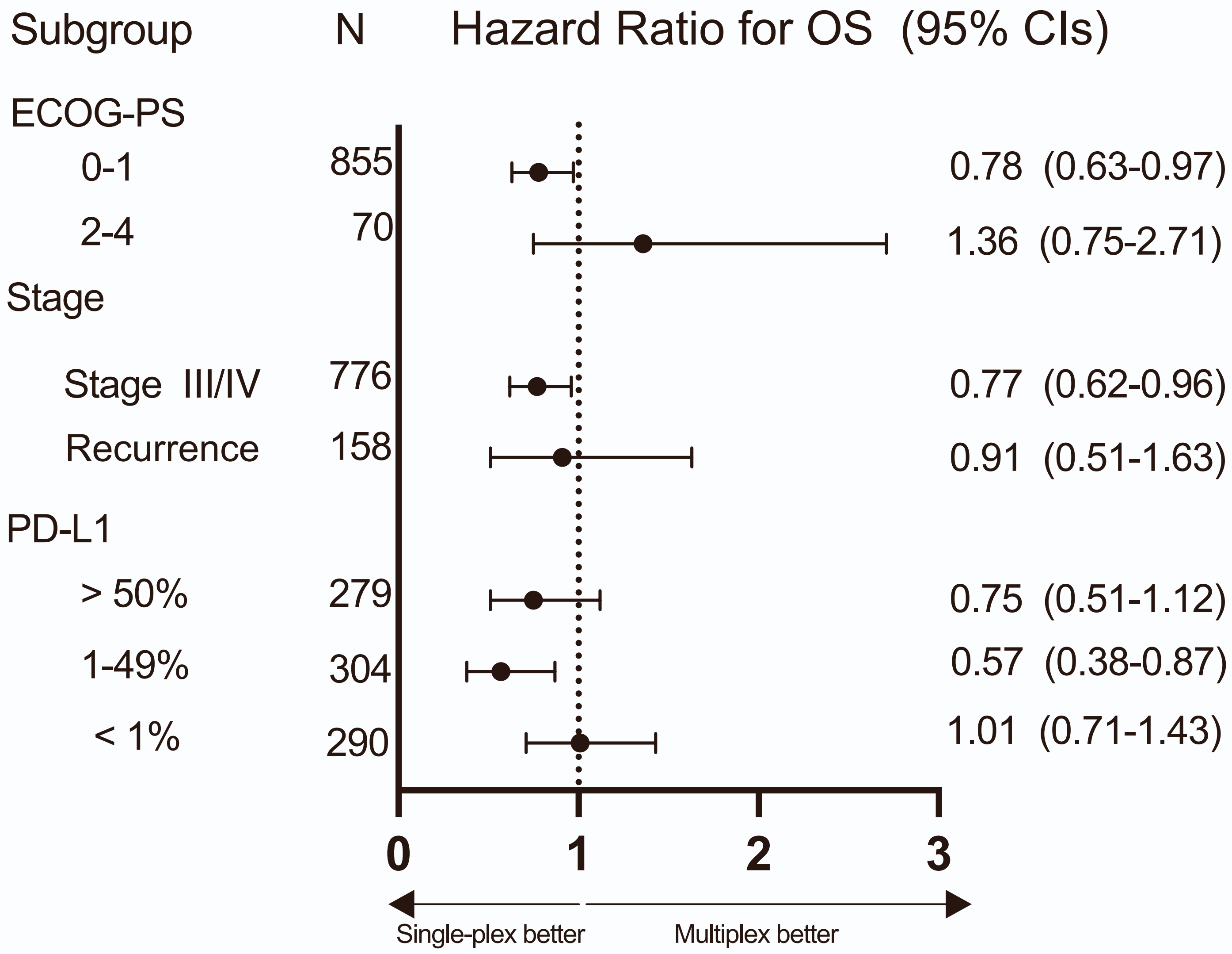

OS: overall survival, ECOG-PS: Eastern Cooperative Oncology Group performance status, PD-L1: programmed death-ligand 1, HRs: hazard ratios, CIs: confidence intervals.

Figure S9. Kaplan-Meier curves for OS of the patients (A) who were evaluated for *EGFR* mutations, (B) who were excluded for *EGFR* mutations, (C) who received ICI-containing regimens as first-line therapy, and (D) who received molecular targeted therapy at any line.

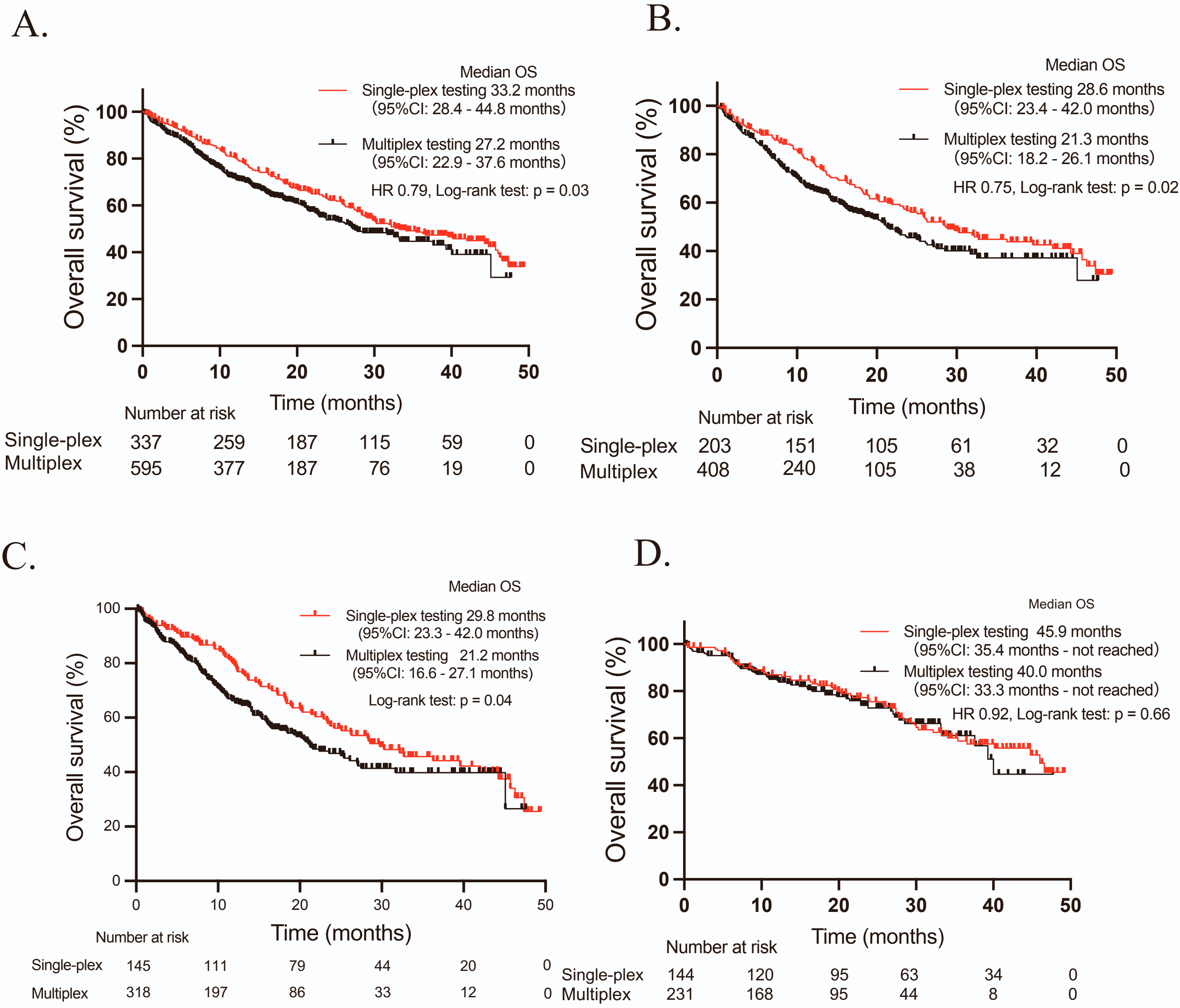

OS: overall survival, *EGFR*: epidermal growth factor receptor, ICI: immune checkpoint inhibitor, CIs: confidence intervals.

Figure S10. Kaplan-Meier curves for OS of (A) patients who received EGFR-TKIs as first-line therapy, (B) patients who received osimertinib, and (C) patients who received EGFR-TKIs excluding osimertinib, compared with biomarker testing modality.
Kaplan-Meier curves for OS of (D) patients who received first or third EGFR-TKIs as first-line therapy, (E) patients with single-plex group, and (F) patients with multiplex group, compared with EGFR-TKI generation (first vs. third).

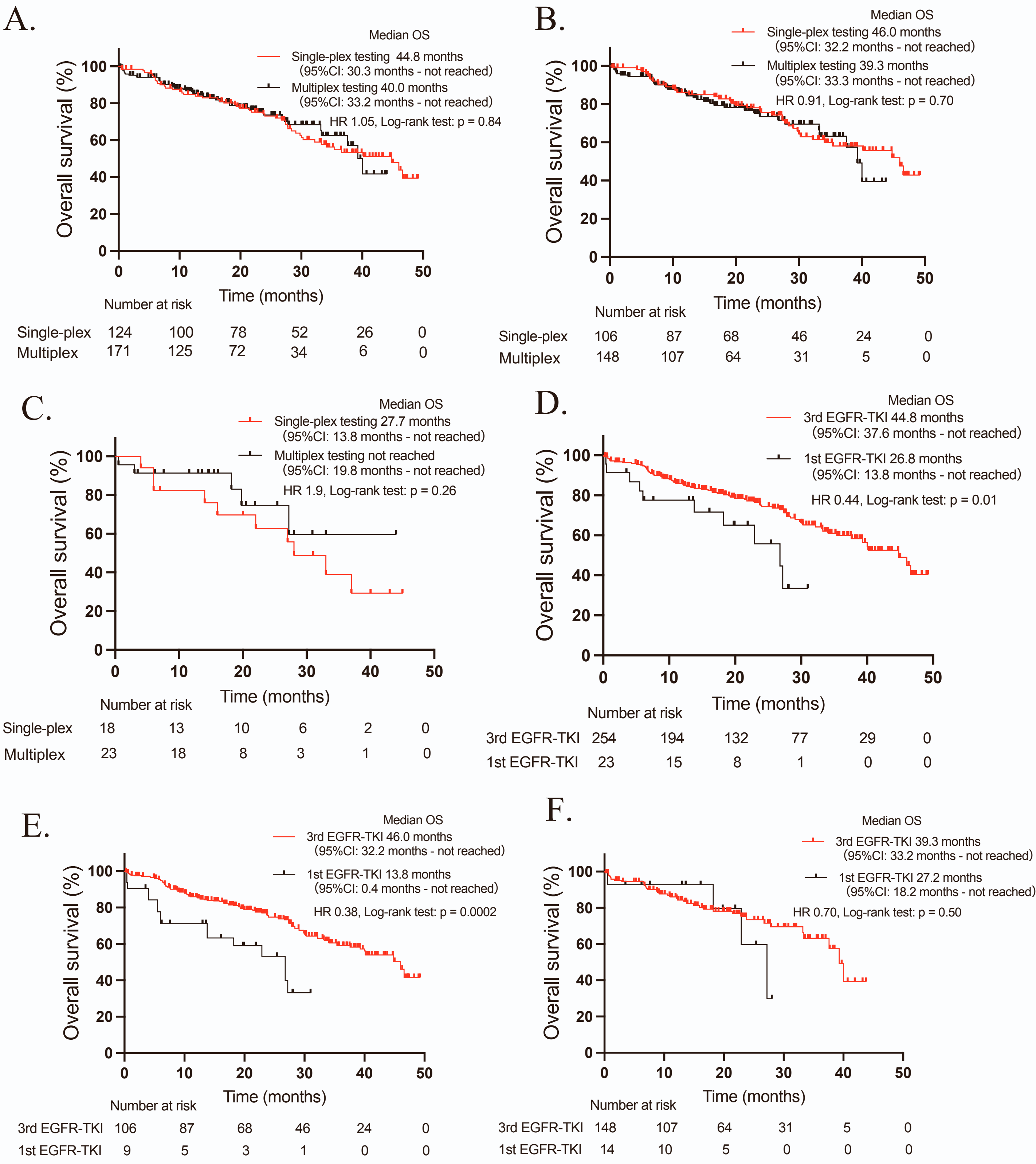

OS: overall survival, *EGFR*: epidermal growth factor receptor, TKIs: tyrosine kinase inhibitors, CI: confidence interval .

Figure S11. Kaplan-Meier curves for OS of (A) all patients harboring actionable driver alterations excluding *EGFR* or *ALK* and (B) patients stratified by matched therapy status.

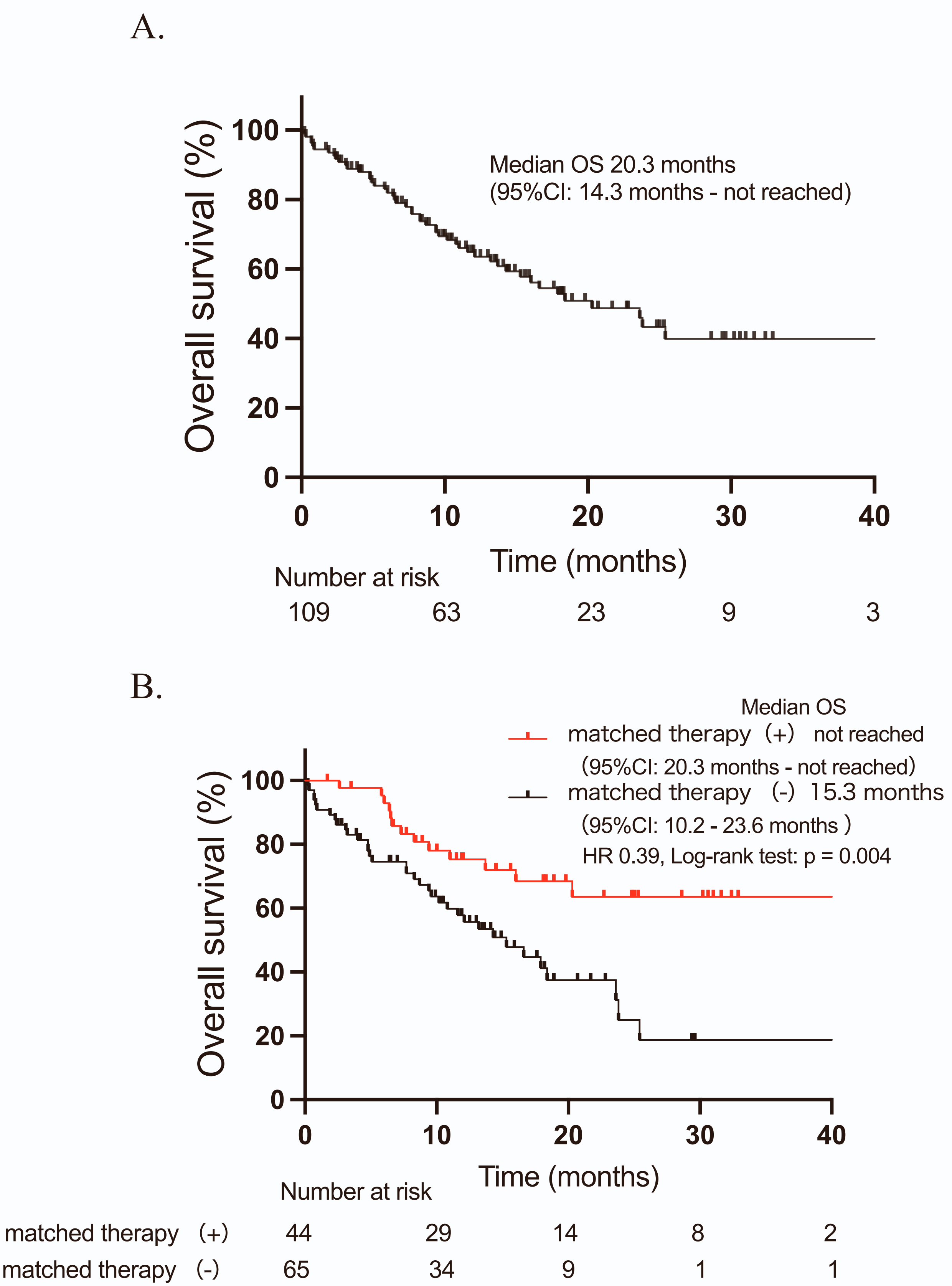

OS: overall survival, *EGFR*: epidermal growth factor receptor, *ALK*: anaplastic lymphoma kinase, CI: confidence interval.

Figure S12. Clinical workflow from diagnostic evaluation to treatment initiation in patients with suspected non-small cell lung cancer.

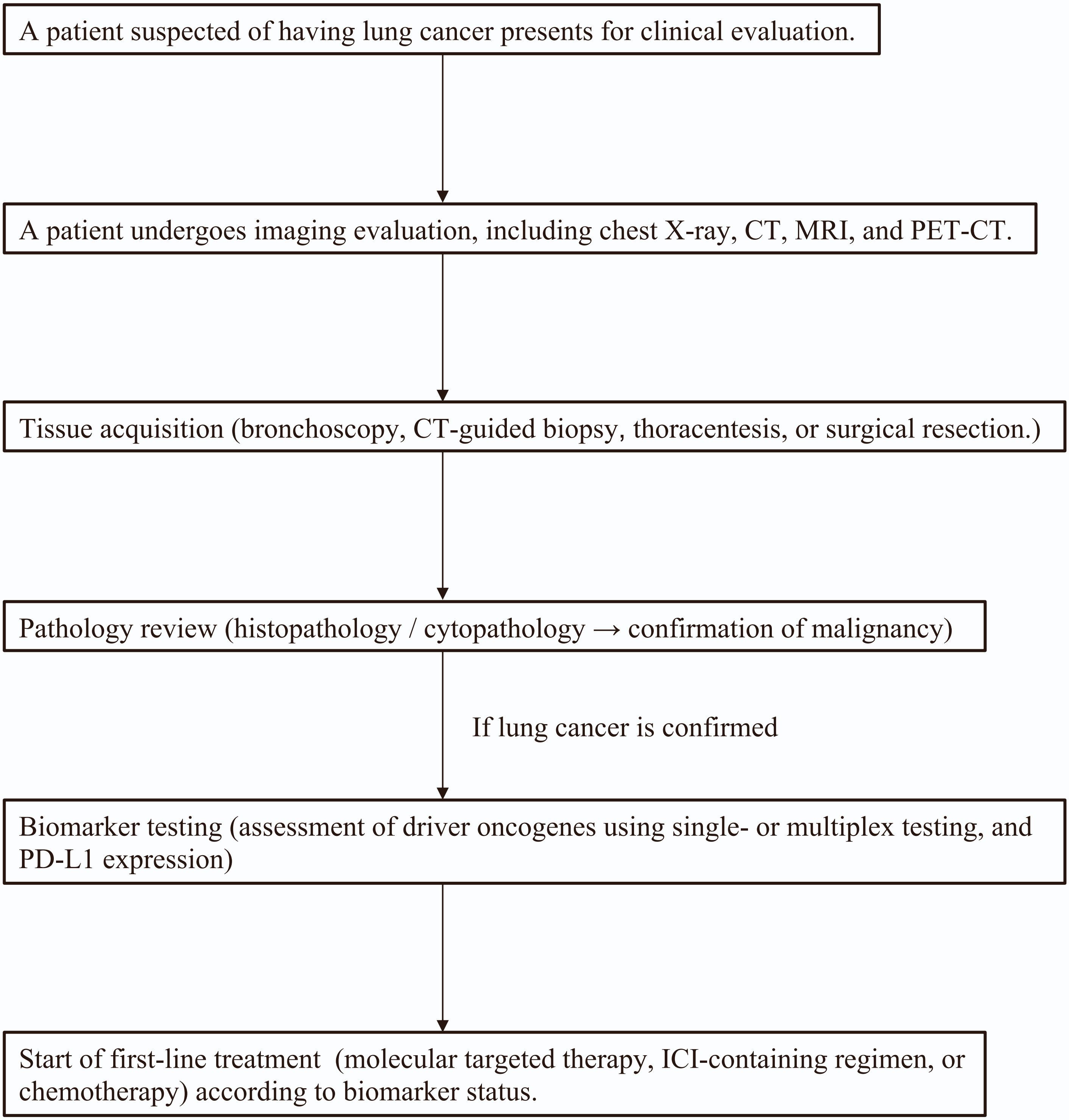

CT: computed tomography, MRI: magnetic resonance imaging, PET-CT: positron emission tomography computed tomography, PD-L1: programmed death-ligand 1, ICI: immune checkpoint inhibitor.

Table S1. Institutional distribution of testing modalities and assay types used in single-plex and multiplex testing, in which (A) summarizes the use of single-plex and multiplex testing across the 12 participating institutions, (B) lists the assay types used for single-plex testing, and (C) lists the assay types used for multiplex testing.

A.

Institutions	Single-plex testing group (n = 339)	Multiplex testing group (n = 595)
No.1 (n = 19)	-	19 (100%)
No. 2 (n = 59)	37 (62.7%)	22 (37.3%)
No. 3 (n = 48)	6 (12.5%)	42 (87.5%)
No. 4 (n = 73)	23 (31.5%)	50 (68.5%)
No. 5 (n = 95)	59 (62.1%)	36 (37.9%)
No. 6 (n = 27)	11 (40.7%)	16 (59.3%)
No. 7 (n = 88)	14 (15.9%)	74 (84.1%)
No. 8 (n = 98)	37 (37.8%)	61 (62.2%)
No. 9 (n = 146)	5 (3.4%)	141 (96.6%)
No. 10 (n = 235)	121 (51.5%)	114 (48.5%)
No. 11 (n = 19)	14 (73.7%)	5 (26.3%)
No. 12 (n = 27)	12 (44.4%)	15 (55.6%)

B.

	Samples (n = 339)
Single-plex testing	
Cobas *EGFR* Mutation Test v2	323 (95.3%)
PNA-LNA PCR clamp (*EGFR*)	16 (4.7%)

C.

	Samples (n = 595)
Multiplex testing	
Oncomine Dx Target Test	434 (72.9%)
i-densy	77 (12.9%)
Amoy 9-in-1 panel	63 (10.6%)
LC-SCRUM Japan program	21 (3.5%)

EGFR: epidermal growth factor receptor, *PCR*: polymerase chain reaction.

Table S2. Detailed distribution of first-line treatment regimens compared with the type of biomarker testing, including *EGFR*-TKIs, ICI-containing regimens, and chemotherapy.

Characteristics	All patients (n = 934)	Single-plex testing group (n = 339)	Multiplex testing group (n = 595)	*p*-Value
Type of first line treatment				
EGFR-TKIs regimens	295 (31.6%)	124 (36.6%)	171 (28.7%)	**0.02**
Non-osimertinib EGFR-TKIs regimens	41 (13.9%)	18 (14.5%)	23 (13.5%)	0.38
Gefitinib	4 (0.4%)	3 (0.9%)	1 (0.2%)	
Erlotinib	1 (0.1%)	1 (0.3%)	-	
Erlotinib plus ramucirumab	16 (1.7%)	4 (1.2%)	12 (2.0%)	
Afatinib	15 (1.6%)	5 (1.5%)	10 (1.7%)	
Dacomitinib	5 (0.5%)	5 (1.5%)	-	
Osimertinib	254 (27.2%)	106 (31.3%)	148 (24.9%)	**0.04**
ICI-containing regimen	463 (49.6%)	145 (42.8%)	318 (53.4%)	**0.002**
PD-1/PD-L1 monotherapy	81 (8.7%)	28 (8.3%)	53 (8.9%)	
PD-1/PD-L1 plus chemotherapy	283 (30.3%)	106 (31.3%)	177 (29.7%)	
PD-1/CTLA inhibitors	23 (2.5%)	5 (1.5%)	18 (3.0%)	
PD-1/CTLA inhibitors plus chemotherapy	40 (4.3%)	2 (0.6%)	38 (6.4%)	
PD-1/PD-L1 inhibitors, chemotherapy plus anti-VEGF antibody	36 (3.9%)	4 (1.2%)	32 (5.4%)	
Chemotherapy	123 (13.2%)	54 (15.9%)	69 (11.9%)	0.09

EGFR: epidermal growth factor receptor, TKIs: tyrosine kinase inhibitors, ICI: immune checkpoint inhibitor, PD-1: programmed cell death protein 1, PD-L1: programmed death-ligand 1, CTLA: cytotoxic T-lymphocyte–associated antigen, VEGF: vascular endothelial growth factor.

Table S3. Number of non-*EGFR*/*ALK* driver oncogenes and proportion of patients who received matched targeted therapies after drug approval in the multiplex testing group.

Driver oncogenes	Number of patients after approval of targeted therapies (n)	Proportion of patients who received targeted therapies	First approval drug	Date of first approval
ROS1 fusion	6	83.3% (n = 5)	Crizotinib	Aug 2017
BRAF V600E	6	100% (n = 6)	Dabrafenib + Trametinib	Mar 2018
MET exon 14 skipping	24	66.7% (n = 16)	Tepotinib	Mar 2020
RET fusion	4	50.0% (n = 2)	Selpercatinib	Sep 2021
KRAS G12C	23	30.4% (n = 7)	Sotorasib	Jan 2022
HER2	0	NA	Trastuzumab deruxtecan	Aug 2023

EGFR: epidermal growth factor receptor, *ALK*: anaplastic lymphoma kinase, *ROS1*: c-ros oncogene 1, *BRAF*: v-raf mouse sarcoma virus tumor gene homolog B1, *RET:* rearranged during transfection, *KRAS:* Kirsten rat sarcoma virus, *HER2:* human epidermal growth factor receptor type 2.

Table S4. Patient and sample characteristics adjusted by propensity score matching.

Characteristics	Single-plex testing group (n = 339)	Multiplex testing group (n = 339)	*p*-value
Age			
Median (range)	70 (32-93)	70 (30-93)	0.62
≥ 75	121 (35.7%)	121 (35.7%)	1.00
Sex			
Female	131 (38.6%)	131 (38.6%)	1.00
ECOG-PS			
0–1	319 (94.1%)	319 (94.1%)	
2–4	19 (5.6%)	17 (5.0%)	0.73
missing data	1 (0.3%)	3 (0.9%)	
Tumor histology			
Adenocarcinoma	289 (85.3%)	285 (84.1%)	1.00
Other	50 (14.7%)	54 (15.9%)	
Stage			
III/IV	289 (85.3%)	289 (85.3%)	1.00
Postoperative Recurrence	50 (14.7%)	50 (14.7%)	
Metastatic site			
Extrathoracic	201 (59.3%)	198 (58.4%)	0.82
Bone	109 (32.3%)	117 (34.5%)	0.51
Brain	75 (22.1%)	87 (25.7%)	0.27
Liver	34 (10.0%)	41 (12.1%)	0.39
PD-L1 TPS			
≥50%	103 (30.4%)	103 (30.4%)	
1–49%	91 (26.8%)	126 (37.2%)	1.00
<1%	106 (31.3%)	95 (28.0%)	
Unknown	39 (11.5%)	15 (4.4%)	
Smoking status			
Current/Former	208 (61.4%)	213 (62.8%)	
Never	124 (36.6%)	124 (36.6%)	0.81
missing data	7 (2.1%)	2 (0.6%)	

ECOG-PS: Eastern Cooperative Oncology Group performance status, PD-L1: programmed death-ligand 1, TPS: tumor proportion score.

Table S5. Patient characteristics who received ICI-containing regimen (n = 466).

Characteristics	Single-plex group (n = 145)	Multiplex group (n = 318)	*p*-value
Age			
Median (range)	70 (45-82)	71 (36-93)	0.38
≥ 75	40 (27.6%)	103 (32.4%)	0.33
Sex			
Female	32 (22.1%)	73 (23.0%)	0.91
ECOG-PS			
0-1	135 (93.1%)	293 (92.1%)	
2-4	10 (6.9%)	23 (7.2%)	0.85
missing data	-	2 (0.6%)	
Tumor histology			
Adenocarcinoma	118 (81.4%)	264 (83.0%)	0.69
Other	27 (18.6%)	54 (17.0%)	
Stage			
III/IV	124 (85.5%)	276 (86.8%)	0.77
Postoperative recurrence	21 (14.5%)	42 (13.2%)	
Metastatic site			
Bone	45 (31.0%)	104 (32.7%)	0.74
Brain	24 (16.6%)	72 (22.6%)	0.24
Liver	12 (8.1%)	32 (10.1%)	0.59
PD-L1 TPS			
≥ 50%	65 (44.8%)	118 (37.1%)	
1-49%	35 (24.1%)	111 (34.9%)	0.13
< 1%	30 (20.7%)	82 (25.8%)	
Unknown	15 (10.3%)	7 (2.2%)	
Smoking status			
Current/Former	115 (79.3%)	253 (79.6%)	
Never	30 (20.7%)	61 (19.2%)	0.90
missing data	-	4 (1.3%)	
Driver oncogenes detected			
Yes	7 (4.8%)	76 (23.9%)	**< 0.001**
KRAS/*HER2*	1 (0.7%)	58 (18.2%)	

ICI: immune checkpoint inhibitor, ECOG-PS: Eastern Cooperative Oncology Group Performance Status, PD-L1: programmed death-ligand 1, TPS: tumor proportion score, *KRAS:* Kirsten rat sarcoma virus, *HER2:* human epidermal growth factor receptor type 2.

Table S6. Cox proportional hazards analyses of OS: multivariable models stratified by institutions.

Items	OS (Multivariate analysis)	
	HR (95% CI)	p-value
Male	1.00 (0.79-1.27)	1.00
Age, years ≥ 75	1.52 (1.23-1.88)	**< 0.001**
Recurrence	0.95 (0.69-1.31)	0.76
ECOG-PS = 0/1 [a]	0.52 (0.37-0.73)	**< 0.001**
Adenocarcinoma	0.74 (0.56-0.98)	0.04
Brain metastasis	1.22 (0.96-1.56)	0.11
Liver metastasis	1.45 (1.07-1.97)	**0.02**
Bone metastasis	1.67 (1.34-2.08)	**< 0.001**
PD-L1 ≥50% [b]	0.87 (0.69-1.14)	0.35
EGFR mutations detected	0.47 (0.36-0.61)	**< 0.001**
Single-plex testing	0.79 (0.63-0.99)	**0.04**

[a] ECOG-PS 0/1 versus ECOG-PS = 2–4 or missing,
[b] PD-L1 TPS ≥ 50% versus PD-L1 TPS < 49% or unknown.

OS: overall survival, *EGFR*: epidermal growth factor receptor,
ECOG-PS: Eastern Cooperative Oncology Group Performance Status, PD-L1: programmed death-ligand 1.

Table S7. Reportable *EGFR*, *KRAS*, *BRAF*, and *ERBB2* gene variants in non–small cell lung cancer according to single-plex or multiplex CDx testing.

Gene	Exon	COSMIC ID (mutation)	nucleotide changes	amino acid change	Single-plex			Multiplex	
					thera screen EGFR	Cobas EGFR	thera screen KRAS	Oncomine Dx Target Test	Amoy 9-in-1 assay
EGFR	3	COSM1451536	c.322A>G	p.R108G	C	C		B2	C
EGFR	3	COSM21683	c.323G>A	p.R108K	C	C		B2	C
EGFR	7	COSM21686	c.865G>A	p.A289T	C	C		B2	C
EGFR	7	COSM21685	c.866C>A	p.A289D	C	C		B2	C
EGFR	7	COSM21687	c.866C>T	p.A289V	C	C		B2	C
EGFR	12	COSM236671	c.1474A>C	p.S492R	C	C		B2	C
EGFR	12	COSM236670	c.1476C>A	p.S492R	C	C		B2	C
EGFR	15	COSM3412196	c.1793G>C	p.G598A	C	C		B2	C
EGFR	15	COSM21690	c.1793G>T	p.G598V	C	C		B2	C
EGFR	18	COSM12988	c.2125G>A	p.E709K	C	C		A	C
EGFR	18	COSM116882	c.2125G>C	p.E709Q	C	C		C	C
EGFR	18	COSM12428	c.2125_2127delinsCAT	p.E709H	C	C		C	C
EGFR	18	COSM13427	c.2126A>C	p.E709A	C	C		A	C
EGFR	18	COSM13009	c.2126A>G	p.E709G	C	C		A	C
EGFR	18	COSM12371	c.2126A>T	p.E709V	C	C		A	C
EGFR	18	COSM1169617	c.2152C>G	p.L718V	C	C		B2	C
EGFR	18	COSM6503269	c.2153T>A	p.L718Q	C	C		B2	C
EGFR	18	COSM1716255	c.2152C>A	p.L718M	C	C		C	C
EGFR	18	COSM41904	c.2153T>C	p.L718P	C	C		C	C
EGFR	18	COSM6252	c.2155G>A	p.G719S	A	B2		A	A
EGFR	18	COSM6253	c.2155G>T	p.G719C	A	B2		A	A
EGFR	18	COSM18441	c.2154_2155delinsTT	p.G719C	C	C		C	C
EGFR	18	COSM18425	c.2156G>A	p.G719D	C	C		A	C
EGFR	18	COSM6239	c.2156G>C	p.G719A	A	B2		A	A
EGFR	18	COSM13979	c.2170G>A	p.G724S	C	C		B2	C
EGFR	19	COSM12423	c.2214_2231dup	p.I740_K745dup	C	C		C	C
EGFR	19	COSM51504	c.2217_2234dup	p.I740_K745dup	C	C		C	C
EGFR	19	COSM4386694	c.2218_2235dup	p.I740_K745dup	C	C		C	C
EGFR	19	COSM26444	c.2219_2236dup	p.K745_E746insVPVAIK	C	C		C	C
EGFR	19	COSM12404	c.2229_2252delins8	p.E746Nfs*15	C	C		C	C
EGFR	19	COSM87245	c.2231T>C	p.I744T	C	C		B2	C
EGFR	19	COSM9233243	c.2230A>T	p.I744F	C	C		C	C
EGFR	19	COSM28512	c.2230A>V	p.I744V	C	C		C	C
EGFR	19	COSM133186	c.2230_2246delins8	p.I744_E749delinsLKR	C	C		C	C
EGFR	19	COSM85798	c.2230_2249delins5	p.I744_A750delinsVK	C	C		C	C
EGFR	19	COSM28602	c.2232C>G	p.I744M	C	C		C	C
EGFR	19	COSM221565	c.2232_2249del	p.K745_A750del	C	C		C	C
EGFR	19	COSM3734668	c.2232_2249delinsAAA	p.E746_A750del	C	C		C	C
EGFR	19	COSM26038	c.2233_2247delAAGGAATTAAGAGAA	p.K745_E749del	C	A		A	C
EGFR	19	COSM255152	c.2234_2235ins18	p.K745_E746insTPVAIK	C	C		C	C
EGFR	19	COSM1190791	c.2234_2248delAGGAATTAAGAGAAG	p.K745_A750delinsT	C	C		A	C
EGFR	19	COSM1190791	c.2234_2248del	p.K745_A750delinsT	C	C		C	C
EGFR	19	COSM18420	c.2235_2237del	p.E746del	C	C		C	C
EGFR	19	COSM28517	c.2235_2246delGGAATTAAGAGA	p.E746_E749del	C	C		A	C
EGFR	19	COSM13550	c.2235_2248delinsAATTC	p.E746_A759deinsIP	C	A		C	C
EGFR	19	COSM6223	c.2235_2249delGGAATTAAGAGAAGC	p.E746_A750del	A	A		A	A
EGFR	19	COSM13549	c.2235_2251delinsAG	p.E746_T751delinsA	C	C		C	C
EGFR	19	COSM13552	c.2235_2251delinsAATTC	p.E746_T751delinsIP	C	A		C	C
EGFR	19	COSM6506513	c.2235_2251delins8	p.E746_T751delinsFPS	C	C		C	C
EGFR	19	COSM13551	c.2235_2252delGGAATTAAGAGAAGCAACinsAAT	p.E746_T751delinsI	A	A		A	A
EGFR	19	COSM24869	c.2235_2252del	p.E746_T751del	C	C		C	C
EGFR	19	COSM12385	c.2235_2255delinsAAT	p.E746_S752delinsI	C	A		C	C
EGFR	19	COSM3727812	c.2236_2241delins6	p.E746_L747delinsNY	C	C		C	C
EGFR	19	COSM12413	c.2236_2248delins4	p.E746_A750delinsRP	C	C		C	C
EGFR	19	COSM13557	c.2236_2248delins4	p.E746_A750delinsQP	C	C		C	C
EGFR	19	COSM6966471	c.2236_2248delins4	p.E746_A750delinsIP	C	C		C	C
EGFR	19	Su2017	c.2236_2249del	p.E746_A751delins*fs	C	C		C	C
EGFR	19	COSM6225	c.2236_2250delGAATTAAGAGAAGCA	p.E746_A750del	A	A		A	A
EGFR	19	COSM6947327	c.2236_2250delinsCCT	p.E746_A750delinsP	C	C		C	C
EGFR	19	COSM26513	c.2236_2251delinsT	p.E746_T751delinsS	C	C		C	C
EGFR	19	COSM26680	c.2236_2252delinsAT	p.E746_T751delinsI	C	C		C	C
EGFR	19	COSM22999	c.2236_2252delinsCA	p.E746_T751delinsQ	C	C		C	C
EGFR	19	COSM133187	c.2236_2252delinsCT	p.E746_T751delinsL	C	C		C	C
EGFR	19	COSM12728	c.2236_2253delGAATTAAGAGAAGCAACA	p.E746_T751del	A	A		A	A
EGFR	19	COSM51526	c.2236_2253delins6	p.E746_T751delinsIP	C	C		C	C
EGFR	19	COSM133188	c.2236_2255delinsAT	p.E746_S752delinsI	C	C		C	C
EGFR	19	COSM133189	c.2236_2256del	p.E746_S752del	C	C		C	C
EGFR	19	COSM133190	c.2236_2256delinsATC	p.E746_S752delinsI	C	C		C	C
EGFR	19	COSM133191	c.2236_2257delins4	p.E746_P753delinsIS	C	C		C	C
EGFR	19	COSM13200	c.2236_2257delins4	p.E746_P753delinsLS	C	C		C	C
EGFR	19	COSM144207	c.2237_2248delinsCAC	p.E746_A750delinsAP	C	C		C	C
EGFR	19	COSM28623	c.2237_2250delins5	p.E746_A750delinsVP	C	C		C	C
EGFR	19	COSM12678	c.2237_2251delAATTAAGAGAAGCAA	p.E746_T751delinsA	A	A		A	A
EGFR	19	COSM18421	c.2237_2251delinsTTC	p.E746_T751delinsVP	C	C		C	C
EGFR	19	COSM53205	c.2237_2251delinsTGG	p.E746_T751delinsVA	C	C		C	C
EGFR	19	COSM6924852	c.2237_2251delins6	p.E746_T751delinsAPS	C	C		C	C
EGFR	19	COSM6968288	c.2237_2251delinsTCC	p.E746_T751delinsVP	C	C		C	C
EGFR	19	COSM6980200	c.2237_2251delinsTTT	p.E746_T751delinsVS	C	C		C	C
EGFR	19	COSM12386	c.2237_2252delinsT	p.E746_T751delinsV	C	A		C	C
EGFR	19	COSM12416	c.2237_2253delAATTAAGAGAAGCAACAinsTTGCT	p.E746_T751delinsVA	C	A		A	C
EGFR	19	COSM133193	c.2237_2253delinsTC	p.E746_T751delinsV	C	C		C	C
EGFR	19	COSM52935	c.2237_2253delins5	p.E746_T751delinsVP	C	C		C	C
EGFR	19	COSM12367	c.2237_2254del18	p.E746_S752>A	A	A		C	A
EGFR	19	COSM12384	c.2237_2255delAATTAAGAGAAGCAACATCinsT	p.E746_S752delinsV	A	A		A	A
EGFR	19	COSM18426	c.2237_2256delinsTC	p.E746_S752delinsV	C	C		C	C
EGFR	19	COSM674057	c.2237_2256delinsTG	p.E746_S752delinsV	C	C		C	C
EGFR	19	COSM18427	c.2237_2257delinsTCT	p.E746_P753delinsVS	C	A		C	C
EGFR	19	COSM51524	c.2237_2258delins4	p.E746_P753delinsVQ	C	C		C	C
EGFR	19	COSM6978341	c.2238_2247del	p.L747Qfs*16	C	C		C	C
EGFR	19	COSM12422	c.2238_2248delATTAAGAGAAGinsGC	p.L747_A750delinsP	A	A		A	A
EGFR	19	COSM18428	c.2238_2248delinsTC	p.E746_A750delinsDP	C	C		C	C
EGFR	19	COSM6974307	c.2238_2250delinsC	p.E746_A750delinsD	C	C		C	C
EGFR	19	COSM22944	c.2238_2251delinsGC	p.L747_T751del insP	C	C		C	C
EGFR	19	COSM23571	c.2238_2252del15	p.L747_T751del	C	A		C	A
EGFR	19	COSM12419	c.2238_2252delATTAAGAGAAGCAACinsGCA	p.L747_T751delinsQ	A	A		A	A
EGFR	19	COSM12421	c.2238_2255delins6	p.L747_S752delinsQH	C	C		C	C

Gene	Exon	COSMIC ID (mutation)	nucleotide changes	amino acid change	Single-plex			Multiplex	
					thera screen EGFR	Cobas EGFR	thera screen KRAS	Oncomine Dx Target Test	Amoy 9-in-1 assay
EGFR	19	COSM6220	c.2238_2255delATTAAGAGAAGCAACATC	p.E746_S752delinsD	A	A		A	A
EGFR	19	COSM26441	c.2238_2256delins4	p.L747_S752delinsQ	C	C		C	A
EGFR	19	COSM255211	c.2238_2258del	p.L747_P753del	C	C		C	C
EGFR	19	—	c.2238_2261delinsGCAACATCT	p.E746_K754delinsEQHL	C	C		C	A
EGFR	19	COSM24267	c.2239_2240delinsCC	p.L747P	C	C		C	C
EGFR	19	COSM6218	c.2239_2247delTTAAGAGAA	p.E746_R748del	A	A		A	A
EGFR	19	COSM6952818	c.2239_2247delinsC	p.L747Rfs*13	C	C		C	C
EGFR	19	COSM12382	c.2239_2248delTTAAGAGAAGinsC	p.L747_A750delinsP	A	A		A	A
EGFR	19	COSM4170220	c.2239_2250delinsCCG	p.L747_A750delinsP	C	C		C	C
EGFR	19	COSM9179903	c.2239_2250del	p.L747_A750del	C	C		C	C
EGFR	19	—	c.2239_2250delinsCCC	p.L747_A750delinsP	C	C		C	A
EGFR	19	COSM12383	c.2239_2251delTTAAGAGAAGCAAinsC	p.L747_T751delinsP	A	A		A	A
EGFR	19	COSM12420	c.2239_2252delinsCA	p.L747_T751delinsQ	C	C		C	C
EGFR	19	COSM23572	c.2239_2253delinsGCT	p.L747_T751delinsA	C	C		C	C
EGFR	19	COSM6254	c.2239_2253del15	p.L747_T751del	A	A		C	A
EGFR	19	COSM51503	c.2239_2253delinsAAT	p.L747_T751delinsN	C	C		C	C
EGFR	19	COSM133196	c.2239_2255delins5	p.L747_S752delinsQH	C	C		C	C
EGFR	19	COSM6255	c.2239_2256delTTAAGAGAAGCAACATCT	p.L747_S752del	A	A		A	A
EGFR	19	COSM12403	c.2239_2256delinsCAA	p.L747_S752delinsQ	C	A		C	A
EGFR	19	COSM12387	c.2239_2258delTTAAGAGAAGCAACATCTCCinsCA	p.L747_P753delinsQ	A	A		A	A
EGFR	19	COSM51501	c.2239_2259delinsCAA	p.L747_P753delinsQ	C	C		C	C
EGFR	19	—	c.2239_2261delinsCAATT	p.L747_K754delinsQL	C	C		C	A
EGFR	19	COSM1667023	c.2239_2261delins11	p.L747_K754delinsANKG	C	C		C	C
EGFR	19	COSM24970	c.2239_2262del	p.L747_K754del	C	C		C	C
EGFR	19	COSM85891	c.2239_2264delins5	p.L747_A755delinsAN	C	C		C	C
EGFR	19	COSM7410537	c.2240delins12	p.L747*	C	C		C	C
EGFR	19	COSM4170221	c.2240_2248del	p.L747_A750delinsS	C	C		C	C
EGFR	19	COSM6210	c.2240_2251delTAAGAGAAGCAA	p.L747_T751delinsS	A	A		A	A
EGFR	19	COSM12369	c.2240_2254delTAAGAGAAGCAACAT	p.L747_T751del	A	A		A	A
EGFR	19	COSM12370	c.2240_2257delTAAGAGAAGCAACATCTC	p.L747_P753delinsS	A	A		A	A
EGFR	19	COSM20883	c.2240_2261delins4	p.L747_K754delinsST	C	C		C	C
EGFR	19	COSM6933365	c.2240_2263delins6	p.L747_A755delinsSMS	C	C		C	C
EGFR	19	COSM1667026	c.2240_2264delins7	p.L747_A755delinsSKG	C	C		C	C
EGFR	19	COSM18442	c.2241_2244delins4	p.L747_R748delinsFP	C	C		C	C
EGFR	19	COSM26440	c.2248_2273delinsCC	p.A750_E758delinsP	C	C		C	C
EGFR	19	COSM26439	c.2248_2274del	p.A750_E758del	C	C		C	C
EGFR	19	COSM5023004	c.2248_2276delins5	p.A750_I759delinsPT	C	C		C	C
EGFR	19	COSM5023005	c.2249_2277delins5	p.A750_I759delinsGS	C	C		C	C
EGFR	19	COSM26718	c.2250_2264del	p.T751_A755del	C	C		C	C
EGFR	19	COSM1667024	c.2250_2276delinsCAA	p.T751_I759delinsN	C	C		C	C
EGFR	19	COSM22945	c.2251_2277delinsTCT	p.T751_I759delinsS	C	C		C	C
EGFR	19	COSM133200	c.2251_2276delinsTC	p.T751_I759delinsS	C	C		C	C
EGFR	19	COSM23634	c.2252_2275del	p.T751_E758del	C	C		C	C
EGFR	19	COSM12410	c.2252_2275delinsG	p.T751Sfs*4	C	C		C	C
EGFR	19	COSM96856	c.2252_2276delinsA	p.T751_I759delinsN	C	C		C	C
EGFR	19	COSM1667027	c.2252_2276delinsG	p.T751_I759delinsS	C	C		C	C
EGFR	19	COSM24270	c.2252_2277delinsAT	p.T751_I759delinsN	C	C		C	C
EGFR	19	COSM22956	c.2252_2277delins8	p.T751_I759delinsREA	C	C		C	C
EGFR	19	COSM6978342	c.2253_2257del	p.S752Efs*9	C	C		C	C
EGFR	19	COSM13556	c.2253_2276del	p.S752_I759del	C	A		C	C
EGFR	19	COSM6256	c.2254_2277del	p.S752_I759del	C	C		C	C
EGFR	19	COSM24269	c.2258_2278del	p.P753_I759del	C	C		C	C
EGFR	19	COSM24972	c.2268_2270dup	p.N756dup	C	C		C	C
EGFR	20	COSM26720	c.2284-5_2290dup	p.A763_Y764insFQEA	C	C		A	C
EGFR	20	COSM1651740	c.2301_2302insTACGTGATG	p.A767_S768insYVM	C	C		A	C
EGFR	20	COSM12425	c.2302_2303insCGCTGGCCA	p.M766_A767insATL	C	C		A	C
EGFR	20	COSM13559	c.2302_2303insTAGCCA	p.M766_A767insAI	C	C		A	C
EGFR	20	COSM1651741	c.2303_2304insTGTGGCCAA	p.S768_V769insVAN	C	C		A	C
EGFR	20	COSM20884	c.2303_2304insTGTGGCCAG	p.M766_A767insASV	C	C		A	C
EGFR	20	COSM6984779	c.2303_2305delGCGinsTCC	p.S768_V769delinsIL	C	C		A	C
EGFR	20	COSM85750	c.2303_2305delGCGinsTCT	p.S768_V769delinsIL	C	C		A	C
EGFR	20	COSM6241	c.2303G>T	p.S768I	A	B2		A	A
EGFR	20	COSM28638	c.2307_2308insATGGCCAGCGTGGAC	p.V769_D770insMASVD	C	C		A	C
EGFR	20	COSM12379	c.2307_2308insTGCGTG	p.S768_V769insVC	C	C		A	C
EGFR	20	COSM20885	c.2307_2308insGACAACGTG	p.N771_P772insVDN	C	C		A	A
EGFR	20	—	c.2308_2308delGinsAACAACCCCC	p.D770delinsNNPH	C	C		A	C
EGFR	20	COSM12376	c.2308_2309insCCAGCGTGG	p.M766_A767insASV	A	B2		A	A
EGFR	20	COSM6983510	c.2308_2309insGCACAC	p.D770delinsGTH	C	C		A	C
EGFR	20	COSM18429	c.2308_2309insGCAGCGTGG	p.A767_S768insSVG	C	C		A	A
EGFR	20	COSM1235344	c.2308_2309insGGAGCGTGG	p.A767_S768insSVG	C	C		A	C
EGFR	20	—	c.2308_2309insGGGGGG	p.V769_D770insGG	C	C		A	C
EGFR	20	COSM18430	c.2308_2309insGGGTCGTGG	p.S768_V769insVGV	C	C		A	C
EGFR	20	COSM12427	c.2308_2309insGTT	p.D770delinsGY	C	C		A	A
EGFR	20	COSM6506514	c.2308_2309insTGG	p.S768_V769insV	C	C		A	C
EGFR	20	—	c.2308_2309insGCACCGTGG	p.S768_V769insVGT	C	C		A	C
EGFR	20	—	c.2309_2309delAinsCCAACCCCCC	p.D770delinsANPP	C	C		A	C
EGFR	20	—	c.2309_2310delACinsGTCCA	p.D770delinsGP	C	C		A	C
EGFR	20	COSM13558	c.2309_2310delACinsCCAGCGTGGAT	p.A767_V769dup	C	B2		A	A
EGFR	20	COSM1651742	c.2309_2310insGCGTGGAGA	p.V769_D770insERG	C	C		A	C
EGFR	20	—	c.2309_2311delACAinsCTGGCC	p.D770_N771delinsAGH	C	C		A	C
EGFR	20	COSM12737	c.2309_2312delACAAinsCTGGTGG	p.D770_N771delinsAGG	C	C		A	C
EGFR	20	—	c.2310_2311insCAC	p.D770_N771insH	C	C		A	C
EGFR	20	COSM4970107	c.2310_2311insCAGCGTGGC	p.D770_N771insQRG	C	C		A	C
EGFR	20	COSM20886	c.2310_2311insGCACCGTGG	p.D770_N771insAPW	C	C		A	C
EGFR	20	COSM13004	c.2310_2311insGGC	p.D770_N771insG	C	C		A	A
EGFR	20	COSM1238029	c.2310_2311insGGCACA	p.D770_N771insGT	C	C		A	A
EGFR	20	COSM22955	c.2310_2311insGGCGAC	p.V769_D770insDG	C	C		A	C
EGFR	20	—	c.2310_2311insGGG	p.D770_N771insG	C	C		A	A
EGFR	20	COSM85795	c.2310_2311insGGGGAC	p.V769_D770insDG	C	C		A	A
EGFR	20	COSM48921	c.2310_2311insGGGTTA	p.D770_N771insGL	C	C		A	A
EGFR	20	COSM655155	c.2310_2311insGGGTTT	p.D770_N771insGF	C	C		A	A
EGFR	20	COSM12378	c.2310_2311insGGT	p.D770_N771insG	A	B2		A	A
EGFR	20	COSM1238030	c.2310_2311insTAC	p.D770_N771insY	C	C		A	C
EGFR	20	COSM6962256	c.2310_2311insTACGTGATGGCCAGCGTGGAC	p.A763_Y764insYVMASV D	C	C		A	C
EGFR	20	—	c.2310_2311insCCA	p.D770_N771insP	C	C		A	C
EGFR	20	—	c.2310_2311insGGCAAC	p.D770_N771insGN	C	C		A	C
EGFR	20	—	c.2311_2311delAinsCCCC	p.N771delinsPH	C	C		A	C
EGFR	20	COSM53189	c.2311_2311delAinsGGTT	p.N771delinsGY	C	C		A	C
EGFR	20	COSM5023007	c.2311_2311delAinsGTCC	p.N771delinsVH	C	C		A	C
EGFR	20	COSM18431	c.2311_2312delAAinsGGGTT	p.N771delinsGF	C	C		A	A

Gene	Exon	COSMIC ID (mutation)	nucleotide changes	amino acid change	Single-plex			Multiplex	
					thera screen EGFR	Cobas EGFR	thera screen KRAS	Oncomine Dx Target Test	Amoy 9-in-1 assay
EGFR	20	COSM22946	c.2311_2312insCAC	p.N771delinsTH	C	C		A	C
EGFR	20	COSM5023008	c.2311_2312insCCA	p.D770_N771insT	C	C		A	C
EGFR	20	COSM6920147	c.2311_2312insGCACCC	p.N771delinsSTH	C	C		A	C
EGFR	20	COSM1651743	c.2311_2312insGCGTCGAAA	p.D770_N771insSVE	C	C		A	C
EGFR	20	COSM13428	c.2311_2312insGCGTGGACA	p.A767_S768insSVD	C	B2		A	A
EGFR	20	COSM24434	c.2311_2312insGTC	p.N771delinsSH	C	C		A	C
EGFR	20	COSM1651744	c.2311_2312insGTGGCC	p.N771delinsSGH	C	C		A	C
EGFR	20	COSM26719	c.2311_2312insTGGCCACCCCCA	p.D770_N771insMATP	C	C		A	C
EGFR	20	—	c.2312_2313insACA	p.N771delinsKH	C	C		A	C
EGFR	20	COSM6438147	c.2312_2313insACT	p.N771delinsKL	C	C		A	A
EGFR	20	—	c.2312_2313insGGACAA	p.V769_D770insDK	C	C		A	C
EGFR	20	COSM13554	c.2312_2315delinsGCGTGGACAACCG	p.N771_P772delinsSVDNR	C	C		A	A
EGFR	20	—	c.2313_2313delCinsGGGG	p.N771delinsKG	C	C		A	C
EGFR	20	COSM13003	c.2313_2314insAAC	p.D770_N771insN	C	C		A	C
EGFR	20	—	c.2313_2314insACA	p.N771_P772insT	C	C		A	C
EGFR	20	COSM6922328	c.2313_2314insGTC	p.N771_P772insV	C	C		A	C
EGFR	20	—	c.2313_2314insTTG	p.N771_P772insL	C	C		A	C
EGFR	20	—	c.2313_2314insACC	p.N771_P772insT	C	C		A	C
EGFR	20	—	c.2314_2315insACAACC	p.D770_N771insNH	C	C		A	C
EGFR	20	COSM1238031	c.2314_2315insACC	p.N771_P772insH	C	C		A	A
EGFR	20	COSM6931207	c.2314_2315insACCACC	p.N771_P772insHH	C	C		A	A
EGFR	20	COSM166390	c.2314_2315insGGCACC	p.N771_P772insRH	C	C		A	C
EGFR	20	—	c.2314_2315insTCC	p.N771_P772insL	C	C		A	C
EGFR	20	COSM48923	c.2315_2316insGACACACCC	p.N771_P772insPTH	C	C		A	C
EGFR	20	COSM6845099	c.2315_2316insGGACAACCC	p.V769_D770insDNP	C	C		A	C
EGFR	20	COSM12388	c.2316_2316delCinsAACCCCT	p.P772_H773insTP	C	C		A	A
EGFR	20	—	c.2316_2316delCinsTCACCCTCACCCT	p.P772_H773insHPHP	C	C		A	C
EGFR	20	—	c.2316_2317insAACCCC	p.D770_N771insNP	C	C		A	C
EGFR	20	COSM6977296	c.2316_2317insACACCCAACCCC	p.D770_N771insNPTP	C	C		A	C
EGFR	20	COSM1651745	c.2316_2317insGACAACCCC	p.V769_D770insDNP	C	C		A	A
EGFR	20	—	c.2316_2317insGGAAACCCC	p.D770_N771insNPG	C	C		A	C
EGFR	20	—	c.2316_2317insGGCAACCCC	p.D770_N771insNPG	C	C		A	C
EGFR	20	—	c.2316_2317insGGCACC	p.P772_H773insGT	C	C		A	C
EGFR	20	—	c.2316_2317insGTGGACAACCCC	p.S768_V769insVDNP	C	C		A	C
EGFR	20	COSM255205	c.2316_2317insGTT	p.P772_H773insV	C	C		A	A
EGFR	20	—	c.2317_2317delCinsAACCCCT	p.H773delinsNPY	C	C		A	C
EGFR	20	—	c.2317_2317delCinsTACAACCCCT	p.H773delinsYNPY	C	C		A	C
EGFR	20	—	c.2317_2317delCinsTACGACCCCAACCCCT	p.H773delinsYDPNPY	C	C		A	C
EGFR	20	—	c.2317_2318insGCC	p.P772_H773insR	C	C		A	C
EGFR	20	—	c.2317_2318insCCAACCCCC	p.D770_N771insNPP	C	C		A	C
EGFR	20	COSM1735761	c.2317_2318insCTAACCCCT	p.H773delinsPNPY	C	C		A	A
EGFR	20	—	c.2317_2318insGTT	p.H773delinsRY	C	C		A	C
EGFR	20	—	c.2318_2319insACA	p.P772_H773insQ	C	C		A	C
EGFR	20	COSM5023006	c.2319_2320insAACCAC	p.P772_H773insHN	C	C		A	C
EGFR	20	COSM12381	c.2319_2320insAACCCCCAC	p.D770_N771insNPH	C	C		A	A
EGFR	20	COSM3727813	c.2319_2320insACACAACCCCCC	p.H773_V774insTQPP	C	C		A	C
EGFR	20	COSM12377	c.2319_2320insCAC	p.P772_H773insH	A	B2		A	A
EGFR	20	COSM131552	c.2319_2320insCAG	p.H773_V774insQ	C	C		A	C
EGFR	20	COSM12380	c.2319_2320insCCCCAC	p.N771_P772insPH	C	C		A	A
EGFR	20	—	c.2319_2320insAACCCCCAT	p.N771_H773dup	C	C		A	C
EGFR	20	—	c.2319_2320insCACCCCCAC	p.N771_P772insPHH	C	C		A	C
EGFR	20	—	c.2319_2320insTAC	p.H773_V774insY	C	C		A	A
EGFR	20	COSM1238028	c.2320_2321insCCCACG	p.P772_H773insHA	C	C		A	A
EGFR	20	COSM51544	c.2320_2321insGCAACCCCCACG	p.D770_N771insNPHG	C	C		A	C
EGFR	20	COSM18432	c.2321_2322insCCACGT	p.P772_H773insHV	C	C		A	A
EGFR	20	—	c.2321_2322insACACGT	p.P772_H773insHV	C	C		A	C
EGFR	20	COSM22948	c.2322_2323insCACGTG	p.P772_H773insHV	C	C		A	A
EGFR	20	COSM4170223	c.2322_2323insCCACGT	p.V774_C775insPR	C	C		A	C
EGFR	20	COSM6845098	c.2322_2323insCCCCACGTG	p.N771_P772insPHV	C	C		A	C
EGFR	20	COSM22954	c.2324G>A	p.C775Y	C	C		B2	C
EGFR	20	COSM6240	c.2369C>T	p.T790M	A	A		A	A
EGFR	20	—	c.2374C>G	p.L792V	C	C		B2	C
EGFR	20	COSM6493934	c.2375T>A	p.L792H	C	C		B2	C
EGFR	20	COSM20891	c.2386G>A	p.G796S	C	C		B2	C
EGFR	20	COSM6493935	c.2386G>C	p.G796R	C	C		B2	C
EGFR	20	—	c.2386G>T	p.G796C	C	C		B2	C
EGFR	20	COSM6493937	c.2389T>A	p.C797S	C	C		B2	A
EGFR	20	COSM5945664	c.2390G>C	p.C797S	C	C		B2	A
EGFR	21	COSM13424	c.2497T>G	p.L833V	C	C		C	C
EGFR	21	COSM13426	c.2512C>G	p.L838V	C	C		C	C
EGFR	21	COSM87246	c.2561C>T	p.T854I	C	C		B2	C
EGFR	21	COSM12366	c.2572C>A	p.L858M	C	C		B2	C
EGFR	21	COSM6224	c.2573T>G	p.L858R	A	A		A	A
EGFR	21	COSM12429	c.2573_2574delinsGT	p.L858R	C	A		C	C
EGFR	21	COSM133630	c.2573_2574delinsGA	p.L858R	C	C		C	C
EGFR	21	COSM13553	c.2572_2573inv	p.L858R	C	C		C	C
EGFR	21	COSM6213	c.2582T>A	p.L861Q	A	B2		A	A
EGFR	21	COSM12374	c.2582T>G	p.L861R	C	C		A	C
BRAF	11	COSM453	c.1397G>A	p.G466E				B2	C
BRAF	11	COSM451	c.1397G>T	p.G466V				B2	C
BRAF	11	COSM457	c.1405G>A	p.G469R				B2	C
BRAF	11	COSM460	c.1406G>C	p.G469A				B2	C
BRAF	11	COSM459	c.1406G>T	p.G469V				B2	C
BRAF	15	COSM27639	c.1780G>A	p.D594N				B2	C
BRAF	15	COSM467	c.1781A>G	p.D594G				B2	C
BRAF	15	COSM469	c.1786G>C	p.G596R				C	C
BRAF	15	COSM7807516	c.1786G>A	p.G596S				C	C
BRAF	15	COSM6936824	c.1786G>T	p.G596C				C	C
BRAF	15	COSM9145962	c.1787G>T	p.G596V				C	C
BRAF	15	COSM26506	c.1787G>A	p.G596D				C	C
BRAF	15	COSM473	c.1798_1799delGTinsAA	p.V600K				B2	C
BRAF	15	COSM474	c.1798_1799delGTinsAG	p.V600R				B2	C
BRAF	15	COSM475	c.1799_1800delTGinsAA	p.V600E				A	C
BRAF	15	COSM1133	c.1799_1801delTGA	p.V600_K601delinsE				B2	C
BRAF	15	COSM476	c.1799T>A	p.V600E				A	A
BRAF	15	COSM478	c.1801A>G	p.K601E				B2	C
KRAS	2	COSM512	c.34_35delGGinsTT	p.G12F			C	B2	C
KRAS	2	COSM517	c.34G>A	p.G12S			C	B2	B2
KRAS	2	COSM518	c.34G>C	p.G12R			C	B2	B2
KRAS	2	COSM516	c.34G>T	p.G12C			A	B2	A

Gene	Exon	COSMIC ID (mutation)	nucleotide changes	amino acid change	Single-plex			Multiplex	
					thera screen EGFR	Cobas EGFR	thera screen KRAS	Oncomine Dx Target Test	Amoy 9-in-1 assay
KRAS	2	COSM521	c.35G>A	p.G12D			C	B2	B2
KRAS	2	COSM522	c.35G>C	p.G12A			C	B2	B2
KRAS	2	COSM520	c.35G>T	p.G12V			C	B2	B2
KRAS	2	COSM528	c.37G>A	p.G13S			C	B2	C
KRAS	2	COSM529	c.37G>C	p.G13R			C	B2	C
KRAS	2	COSM527	c.37G>T	p.G13C			C	B2	B2
KRAS	2	COSM531	c.38_39delGCinsAT	p.G13D			C	B2	C
KRAS	2	COSM532	c.38G>A	p.G13D			C	B2	C
KRAS	2	COSM533	c.38G>C	p.G13A			C	B2	C
KRAS	2	COSM534	c.38G>T	p.G13V			C	B2	C
KRAS	3	COSM546	c.175G>A	p.A59T			C	B2	C
KRAS	3	COSM547	c.176C>A	p.A59E			C	B2	C
KRAS	3	COSM28518	c.176C>G	p.A59G			C	B2	C
KRAS	3	COSM87298	c.180_181delTCinsAA	p.Q61K			C	B2	C
KRAS	3	COSM549	c.181C>A	p.Q61K			C	B2	C
KRAS	3	COSM550	c.181C>G	p.Q61E			C	B2	C
KRAS	3	COSM551	c.182A>C	p.Q61P			C	B2	C
KRAS	3	COSM552	c.182A>G	p.Q61R			C	B2	C
KRAS	3	COSM553	c.182A>T	p.Q61L			C	B2	C
KRAS	3	COSM554	c.183A>C	p.Q61H			C	B2	C
KRAS	3	COSM555	c.183A>T	p.Q61H			C	B2	C
KRAS	4	COSM19940	c.351A>C	p.K117N			C	B2	C
KRAS	4	COSM28519	c.351A>T	p.K117N			C	B2	C
KRAS	4	COSM19404	c.436G>A	p.A146T			C	B2	C
KRAS	4	COSM19905	c.436G>C	p.A146P			C	B2	C
KRAS	4	COSM19900	c.437C>T	p.A146V			C	B2	C
ERBB2	8	COSM94225	c.929C>A	p.S310Y				A	C
ERBB2	8	COSM48358	c.929C>T	p.S310F				A	C
ERBB2	8	COSM6906440	c.929_930delinsAT	p.S310Y				C	C
ERBB2	17	COSM1756937	c.1967C>T	p.S656F				C	C
ERBB2	17	COSM1382870	c.1970C>T	p.A657V				C	C
ERBB2	17	COSM6503261	c.1976T>A	p.V659D				C	C
ERBB2	17	COSM3724566	c.1976_1977inv	p.V659E				C	C
ERBB2	17	COSM6503262	c.1976_1977delinsAG	p.V659E				C	C
ERBB2	17	COSM4849559	c.1978G>C	p.G660R				C	C
ERBB2	17	COSM4681497	c.1979G>A	p.G660D				C	C
ERBB2	17	COSM6986868	c.1981A>G	p.I661V				C	C
ERBB2	17	COSM6352924	c.1984C>G	p.L662V				C	C
ERBB2	17	COSM978667	c.1988T>C	p.L663P				C	C
ERBB2	17	COSM7347803	c.2009T>G	p.V670G				C	C
ERBB2	17	COSM6854377	c.2017A>T	p.I673F				C	C
ERBB2	17	COSM7339427	c.2024T>C	p.I675T				C	C
ERBB2	17	COSM4958454	c.2030G>A	p.R677Q				C	C
ERBB2	17	COSM6965226	c.2032C>T	p.R678W				C	C
ERBB2	17	COSM436498	c.2033G>A	p.R678Q				A	C
ERBB2	18	COSM14059	c.2198C>T	p.T733I				A	C
ERBB2	19	COSM683	c.2263_2264delTTinsCC	p.L755P				A	C
ERBB2	19	COSM6906940	c.2263_2264delTTinsGC	p.L755A				A	C
ERBB2	19	COSM1205571	c.2263T>A	p.L755M				A	C
ERBB2	19	COSM5029269	c.2264_2278delTGAGGGAAAACACAT	p.L755_T759del				B2	C
ERBB2	19	COSM14060	c.2264T>C	p.L755S				A	C
ERBB2	19	COSM436499	c.2264T>G	p.L755W				A	C
ERBB2	19	COSM7706857	c.2299A>T	p.I767F				B2	C
ERBB2	19	COSM51317	c.2301C>G	p.I767M				A	C
ERBB2	19	COSM1302747	c.2305G>A	p.D769N				A	C
ERBB2	19	COSM13170	c.2305G>C	p.D769H				A	C
ERBB2	19	COSM1251412	c.2305G>T	p.D769Y				A	C
ERBB2	20	COSM20959	c.2324_2325insATACGTGATGGC	p.E770_A771insAYVM				A	B2
ERBB2	20	—	c.2325_2326insACCGTGATGGCT	p.Y772_V773insVMAT				A	C
ERBB2	20	COSM12558	c.2325_2326insTACGTGATGGCT	p.A771_Y772insYVMA				A	B2
ERBB2	20	COSM12554	c.2326_2326delGinsCTTT	p.G776delinsLC				A	B2
ERBB2	20	COSM19875	c.2326_2326delGinsTTGT	p.G776delinsLC				A	B2
ERBB2	20	—	c.2326_2327delGGinsTGTGT	p.G776delinsCV				B2	C
ERBB2	20	—	c.2326_2327insTAG	p.A775_G776insV				A	C
ERBB2	20	COSM7774585	c.2326_2327insTAT	p.G776delinsVC				A	B2
ERBB2	20	—	c.2326_2327insTCG	p.A775_G776insV				A	C
ERBB2	20	—	c.2326_2327insTCGTGATGGCTG	p.Y772_V773insVMAV				B2	C
ERBB2	20	COSM85995	c.2326_2327insTCT	p.G776delinsVC				A	B2
ERBB2	20	—	c.2326_2327insTGG	p.A775_G776insV				A	C
ERBB2	20	COSM12553	c.2326_2327insTGT	p.G776delinsVC				A	B2
ERBB2	20	—	c.2326_2327insTTG	p.A775_G776insV				A	C
ERBB2	20	COSM12552	c.2326_2327insTTT	p.G776delinsVC				A	B2
ERBB2	20	COSM685	c.2326G>A	p.G776S				A	C
ERBB2	20	COSM303938	c.2326G>T	p.G776C				A	C
ERBB2	20	COSM20895	c.2326delinsTTAT	p.G776delinsLC				C	B2
ERBB2	20	COSM18609	c.2327G>T	p.G776V				A	C
ERBB2	20	COSM6438151	c.2328_2329insCTT	p.G776_V777insL				A	C
ERBB2	20	COSM14064	c.2329G>A	p.V777M				A	C
ERBB2	20	COSM436500	c.2329G>C	p.V777L				A	C
ERBB2	20	COSM14062	c.2329G>T	p.V777L				A	C
ERBB2	20	—	c.2330_2331insAGGTTGTGT	p.G776_V777insVGC				A	C
ERBB2	20			G776_V777delinsCVCG				C	C
ERBB2	20			G776_V777insVC				C	C
ERBB2	20	COSM303939	c.2331_2332insTGTGGG	p.V777_G778insCG				A	B2
ERBB2	20	COSM5802314	c.2333_2334insGCTCCCCAG	p.G778_S779insLPS				A	C
ERBB2	20	COSM26681	c.2333_2334insGGG	p.V777_G778insG				A	C
ERBB2	20	—	c.2335_2336insGCCCAGGCT	p.V777_G778insGCP				A	C
ERBB2	20	COSM681	c.2336_2337insTGTGGGCTC	p.G776_V777insVGS				A	C
ERBB2	20	—	c.2338_2339insGCTCCC	p.G778_S779insSR				B2	C
ERBB2	20	COSM6865893	c.2339_2340insCGGCTCCCC	p.V777_G778insGSP				A	B2
ERBB2	20	COSM12555	c.2339_2340insGGGCTCCCC	p.V777_G778insGSP				A	B2
ERBB2	20	COSM303948	c.2339_2340insTGGCTCCCC	p.V777_G778insGSP				A	B2
ERBB2	20	COSM12556	c.2340_2341insGGCTCCCCA	p.V777_G778insGSP				A	B2
ERBB2	20			T798I				C	C
ERBB2	21	COSM14065	c.2524G>A	p.V842I				A	C
ERBB2	21	—	c.2584A>G	p.T862A				B2	C
ERBB2	21	—	c.2585C>T	p.T862I				A	C
ERBB2	21	COSM249793	c.2606T>G	p.L869R				A	C
ERBB2	22	COSM14066	c.2686C>T	p.R896C				A	C
ERBB2	22	COSM119971	c.2687G>A	p.R896H				A	C

A: Variants approved for companion diagnostic use. B1: Variants that fall within the scope of drug approval and can theoretically be detected by companion diagnostic tests.
B2: Variants not covered by drug approval but available as reference information. C: Variants that are theoretically undetectable, including those not experimentally confirmed and those presumed to be undetectable by design.

Table S8. Fusion Genes Reportable in non-small cell lung cancer by Oncomine Dx Target Test and Amoy 9-in-1 assay.

Gene	Fusion Gene	Oncomine Dx Target Test	Amoy 9-in-1 assay
ALK	A2M-ALK	A	C
ALK	ATIC-ALK	A	C
ALK	C2orf44-ALK	A	C
ALK	CARS-ALK	A	C
ALK	CLTC-ALK	A	C
ALK	DCTN1-ALK	A	C
ALK	EML4-ALK	A	A
ALK	GTF2IRD1-ALK	A	C
ALK	HIP1-ALK	A	C
ALK	KIF5B-ALK	A	A
ALK	KLC1-ALK	A	A
ALK	PPP4R3B-ALK	A	C
ALK	PRKAR1A-ALK	A	C
ALK	RANBP2-ALK	A	C
ALK	SEC31A-ALK	A	C
ALK	STRN-ALK	A	C
ALK	TFG-ALK	A	A
ALK	TPM1-ALK	A	C
ALK	TPM3-ALK	A	C
ALK	TPM4-ALK	A	C
ALK	TPR-ALK	A	C
ALK	TRAF1-ALK	A	C
ALK	VCL-ALK	A	C
ROS1	CCDC6-ROS1	A	C
ROS1	CD74-ROS1	A	A
ROS1	CEP85L-ROS1	A	C
ROS1	CLIP1-ROS1	A	C
ROS1	CLTC-ROS1	A	C
ROS1	ERC1-ROS1	A	C
ROS1	EZR-ROS1	A	A
ROS1	GOPC-ROS1	A	A
ROS1	HLA_A-ROS1	A	C
ROS1	KDELR2-ROS1	A	C
ROS1	LRIG3-ROS1	A	A
ROS1	MSN-ROS1	A	C
ROS1	KDELR2-ROS1	A	C
ROS1	MYO5A-ROS1	A	C
ROS1	PPFIBP1-ROS1	A	C
ROS1	PWWP2A-ROS1	A	C
ROS1	SDC4-ROS1	A	A
ROS1	SHTN1-ROS1	A	C
ROS1	SLC34A2-ROS1	A	A
ROS1	TFG-ROS1	A	C
ROS1	TPM3-ROS1	A	A
ROS1	ZCCHC8-ROS1	A	C
MET ex14skipping	MET-MET	A	A
RET	ACBD5-RET	A	C
RET	AFAP1-RET	A	C
RET	AKAP13-RET	A	C
RET	CCDC6-RET	A	A
RET	CUX1-RET	A	A
RET	ERC1-RET	A	C
RET	FKBP15-RET	A	C
RET	GOLGA5-RET	A	C
RET	HOOK3-RET	A	C
RET	KIAA1468-RET	A	A
RET	KIF5B-RET	A	A
RET	KIF13A-RET	C	A
RET	KTN1-RET	A	C
RET	MPRIP-RET	C	A
RET	MYO5C-RET	C	A
RET	NCOA4-RET	A	A
RET	PCM1-RET	A	C
RET	PICALM-RET	C	A
RET	PRKAR1A-RET	A	C
RET	RUFY2-RET	A	A
RET	SPECC1L-RET	A	C
RET	TBL1XR1-RET	A	C
RET	TNIP2-RET	C	A
RET	TRIM24-RET	A	C
RET	TRIM27-RET	A	C
RET	TRIM33-RET	A	A
RET	WAC-RET	C	A
NTRK1	BCAN-NTRK1	B2	C
NTRK1	CD74-NTRK1	B2	B2
NTRK1	F11R-NTRK1	C	B2
NTRK1	GRIPAP1-NTRK1	C	B2
NTRK1	IRF2BP2-NTRK1	B2	B2
NTRK1	LMNA-NTRK1	B2	C
NTRK1	MPRIP-NTRK1	B2	B2
NTRK1	NFASC-NTRK1	B2	C
NTRK1	RNF213-NTRK1	B2	C
NTRK1	SQSTM1-NTRK1	B2	B2
NTRK1	SSBP2-NTRK1	B2	B2
NTRK1	TFG-NTRK1	B2	B2
NTRK1	TPM3-NTRK1	B2	B2
NTRK1	TPR-NTRK1	B2	B2
NTRK2	AFAP1-NTRK2	B2	C
NTRK2	AGBL4-NTRK2	B2	C
NTRK2	NACC2-NTRK2	B2	C
NTRK2	SQSTM1-NTRK2	B2	B2
NTRK2	STRN-NTRK2	C	B2
NTRK2	TRIM24-NTRK2	B2	B2
NTRK2	VCL-NTRK2	B2	C
NTRK3	BTBD1-NTRK3	B2	C
NTRK3	COX5A-NTRK3	B2	C
NTRK3	EML4-NTRK3	C	B2
NTRK3	ETV6-NTRK3	B2	B2
NTRK3	RBPMS-NTRK3	C	B2
NTRK3	SQSTM1-NTRK3	C	B2

A: Variants approved for companion diagnostic use. B1: Variants that fall within the scope of drug approval and can theoretically be detected by companion diagnostic tests. B2: Variants not covered by drug approval but available as reference information. C: Variants that are theoretically undetectable, including those not experimentally confirmed and those presumed to be undetectable by design.
